# Phosphorylation of CDK9 at Ser175 Enhances HIV Transcription and Is a Marker of Activated P-TEFb in CD4^+^ T Lymphocytes

**DOI:** 10.1371/journal.ppat.1003338

**Published:** 2013-05-02

**Authors:** Uri R. Mbonye, Giridharan Gokulrangan, Manish Datt, Curtis Dobrowolski, Maxwell Cooper, Mark R. Chance, Jonathan Karn

**Affiliations:** 1 Department of Molecular Biology and Microbiology, Case Western Reserve University School of Medicine, Cleveland, Ohio, United States of America; 2 Center for Proteomics and Bioinformatics, Case Western Reserve University School of Medicine, Cleveland, Ohio, United States of America; Fred Hutchinson Cancer Research Center, United States of America

## Abstract

The HIV transactivator protein, Tat, enhances HIV transcription by recruiting P-TEFb from the inactive 7SK snRNP complex and directing it to proviral elongation complexes. To test the hypothesis that T-cell receptor (TCR) signaling induces critical post-translational modifications leading to enhanced interactions between P-TEFb and Tat, we employed affinity purification–tandem mass spectrometry to analyze P-TEFb. TCR or phorbal ester (PMA) signaling strongly induced phosphorylation of the CDK9 kinase at Ser175. Molecular modeling studies based on the Tat/P-TEFb X-ray structure suggested that pSer175 strengthens the intermolecular interactions between CDK9 and Tat. Mutations in Ser175 confirm that this residue could mediate critical interactions with Tat and with the bromodomain protein BRD4. The S175A mutation reduced CDK9 interactions with Tat by an average of 1.7-fold, but also completely blocked CDK9 association with BRD4. The phosphomimetic S175D mutation modestly enhanced Tat association with CDK9 while causing a 2-fold disruption in BRD4 association with CDK9. Since BRD4 is unable to compete for binding to CDK9 carrying S175A, expression of CDK9 carrying the S175A mutation in latently infected cells resulted in a robust Tat-dependent reactivation of the provirus. Similarly, the stable knockdown of BRD4 led to a strong enhancement of proviral expression. Immunoprecipitation experiments show that CDK9 phosphorylated at Ser175 is excluded from the 7SK RNP complex. Immunofluorescence and flow cytometry studies carried out using a phospho-Ser175-specific antibody demonstrated that Ser175 phosphorylation occurs during TCR activation of primary resting memory CD4+ T cells together with upregulation of the Cyclin T1 regulatory subunit of P-TEFb, and Thr186 phosphorylation of CDK9. We conclude that the phosphorylation of CDK9 at Ser175 plays a critical role in altering the competitive binding of Tat and BRD4 to P-TEFb and provides an informative molecular marker for the identification of the transcriptionally active form of P-TEFb.

## Author Summary

The release of the transcription elongation factor P-TEFb from the 7SK RNP complex and its binding to the HIV Tat transactivator protein enables the efficient transcription of HIV proviruses. In resting memory T-cells, which carry the bulk of the latent HIV viral pool, limiting the cellular levels of P-TEFb ensures that the provirus remains silenced unless the host cell is activated. Here we demonstrate that T-cell receptor (TCR) activation induces phosphorylation of Ser175, a residue which is located at the interface between CycT1, CDK9 and Tat. Phosphorylation of Ser175 occurs on free or 7SK-dissociated P-TEFb and genetic experiments indicate that this modification enhances P-TEFb interaction with Tat resulting in Tat-dependent reactivation of HIV proviral transcription. Modification of Ser175 appears critical for controlling the competitive binding of Tat and the bromodomain protein BRD4 to P-TEFb. Activation of P-TEFb in resting T-cells thus involves both the initial assembly of the 7SK snRNP complex and the subsequent mobilization of P-TEFb by cellular signaling and Tat. Therefore, pSer175 provides an informative molecular marker for the identification of the transcriptionally active form of P-TEFb that can be used to monitor the extent of T-cell activation during therapeutic interventions aimed at virus eradication.

## Introduction

HIV infections persist throughout the lifetimes of patients due to the creation of a latent viral reservoir that is refractory to both antiviral immune responses and antiretroviral therapy (ART) [1], [2], [3], [4], [5]. Genetic and biochemical evidence strongly suggests that the major latent viral reservoir comprises a small population of resting memory CD4^+^ T-cells (∼1 in 10^6^ cells) [6], [7], [8] that are created when effector T-cells acquire a G_o_ resting memory phenotype [9] or when resting memory T-cells become infected [10]. Interruption of ART invariably leads to a rebound of virus production, even in patients that have been suppressed to below detectable levels of viremia for decades [11], [12], [13], [14], [15], [16]. The need to develop novel therapeutic tools to attack the latently infected population is now a widely recognized goal [1], [5], but implementation of this will require both a more detailed understanding of mechanisms underlying proviral latency and the creation of improved analytical tools to monitor the state of the latent proviral reservoir [3], [4], [17].

A key feature that distinguishes HIV transcription from cellular gene transcription and permits efficient entry of proviruses into latency is that it is auto-regulated by the regulatory protein Tat (for reviews see [18], [19], [20], [21], [22]). Because of this feedback mechanism a disproportionate decline in HIV transcription ensues when Tat levels become restricted due to small changes in the efficiency of transcriptional initiation, typically initiated by epigenetic changes to the chromatin structure at the HIV LTR. Epigenetic restriction of the initiation of HIV-1 transcription has been documented in transformed cells [23], [24], [25], [26], *ex vivo* primary cell models for HIV-1 latency [27], [28], [29] and latently infected cells obtained from patients [30]. Additional blocks to HIV-1 transcription initiation found in resting CD4^+^ T-cells and transformed T-cell lines, such as Jurkat cells, include the sequestration of the transcription initiation factors NF-κB [31], [32] and NFAT [33], [34] in the cytoplasm.

In addition to these blocks to transcriptional initiation, Tat-dependent transcriptional elongation in latently infected CD4^+^ T cells is restricted by limiting the availability of the cellular elongation co-factor P-TEFb [35], [36], [37], [38]. P-TEFb is a heterodimer of the CDK9 serine/threonine kinase and a C-type regulatory cyclin, Cyclin T1 (CycT1). Human Cyclin T1 (hCycT1) binds directly to Tat and enhances the co-operative binding of P-TEFb/Tat to TAR RNA by binding to its apical loop [39], [40], [41]. P-TEFb stimulates HIV transcription elongation by phosphorylating a variety of positive and negative factors. Latent HIV proviruses typically carry promoter-proximally paused RNAP II complexes. Hyperphosphorylation of serine residues of the heptad repeats at the CTD of RNAP II by Tat-stimulated P-TEFb enhances its processivity [42], [43], [44], [45]. In addition, phosphorylation of the E-subunit of the negative elongation factor NELF by P-TEFb forces its dissociation from paused RNAP II complexes and allows resumption of productive elongation [46]. Similarly, phosphorylation of the C-terminal region of the SPT5 subunit of DSIF by P-TEFb transforms it into a positive elongation factor [47], [48]. It has also been recently discovered that in addition to stimulating P-TEFb recruitment to the promoter, Tat also mediates the recruitment of a large “super elongation complex” containing numerous additional elongation factors [49], [50], [51], [52].

Despite these overlapping restrictions, T-cell receptor (TCR) stimulation of latently infected T- cells provides all the intracellular signals that are necessary to reactivate productive HIV transcription and is generally regarded as the most efficient way to reactivate latent proviruses [27], [33], [53]. Within minutes of TCR stimulation there is an influx of the transcription initiation factors NF-κB and NFAT into the nucleus [24], [27], [32], [53]. Binding of these transcription factors to the HIV LTR activates chromatin modifying and remodeling events that reverse the epigenetic restrictions associated with proviral latency and the release of paused RNAP II located in the vicinity of the transactivation response element, TAR [24], [53], [54], [55], [56]. Initiation of new rounds of transcription and promoter clearance by RNAP II is then triggered by the recruitment of TFIIH [31], [57] which allows for additional accumulation of paused RNAP II bound by the negative elongation factors NELF and DSIF [58], [59], [60], [61]. The early rounds of transcription lead to the synthesis of additional Tat and a switch to constitutive proviral transcription where RNAP II promoter proximal pausing is continuously overcome by Tat leading to a 100- to 1000-fold increase in the production of viral transcripts.

TCR activation simultaneously stimulates P-TEFb activity by regulating the 7SK snRNP complex. 7SK RNA is a highly conserved 331-nucleotide RNA polymerase III transcript that serves as a platform for the binding of two P-TEFb molecules [62], [63] together with a dimer of the inhibitory proteins HEXIM1 and/or HEXIM2 which maintain CDK9 in a catalytically inactive state [64], [65], [66]. The 7SK snRNP complex also contains the 5′-end capping enzyme MEPCE and the 3′-end uridine-rich binding protein LARP7 that are believed to protect the ends of the RNA from exonuclease cleavage [67], [68].

The molecular mechanisms regulating the binding and release of P-TEFb from the 7SK snRNP are still poorly understood. Incorporation of P-TEFb into 7SK snRNP requires the phosphorylation of CDK9 at Thr186 of its activation loop [69], [70], [71]. This modification is also essential for activating the enzyme's kinase activity [69], [70], [71]. In resting CD4^+^ T lymphocytes CycT1 expression is highly restricted [27], [35], [36], [72] due to translational blocks mediated by miRNA [38], severely limiting the levels of functional P-TEFb in these cells. Activation of CD4^+^ T lymphocytes through the TCR is sufficient to trigger the expression of hCycT1 [36], [73] and the concomitant phosphorylation of Thr186 permitting the assembly of the 7SK snRNP complex.

Tat is able to extract P-TEFb from the 7SK complex by displacing HEXIM1 and inducing conformation changes in the 7SK RNA [74], [75], [76], [77]. When Tat levels are very low, such as during the reactivation of latent proviruses, activation of HIV transcription elongation might be enhanced by a Tat-independent activation signal that triggers the disassembly of 7SK snRNP. Consistent with this hypothesis we recently found that in Jurkat T-cells, TCR activation induces the rapid release of P-TEFb from the nuclear 7SK snRNP complex and enhances its recruitment to the HIV long terminal repeat (LTR) [53]. Fujinaga et al. [78] have recently confirmed that TCR signaling is a potent trigger for P-TEFb dissociation from 7SK RNP. They also found that during TCR mediated disruption of the 7SK RNP complex protein kinase C (PKC) phosphorylates HEXIM1 at Ser158. The Ser158 phosphorylated HEXIM1 protein is unable to bind to 7SK snRNA and is therefore unable to inhibit P-TEFb.

In the experiments to be described we used affinity purification-tandem mass spectrometry (AP-MS/MS) to define key post-translational modifications found on P-TEFb subunits in response to TCR signaling. Our results show that phosphorylation of CDK9 at Ser175 [79] is rapidly induced by TCR signals in memory CD4^+^ T cells. Ser175 phosphorylation occurs subsequent to the dissociation of P-TEFb from 7SK snRNP and this modification plays an important role in controlling the competitive interaction of CDK9 with Tat and the bromodomain protein BRD4, which recruits P-TEFb to cellular genes. These observations are consistent with previous studies showing that certain mutations in Ser175 can reduce P-TEFb interactions with BRD4 [80]. Additionally, using an antibody specific for pSer175 we have shown that phosphorylation of CDK9 at Ser175 provides a sensitive molecular marker for the transcriptionally active form of P-TEFb in primary CD4^+^ T-cells.

## Results

### Affinity purification and tandem mass spectrometry analysis of P-TEFb complexes

In latently infected Jurkat T-cells, TCR-induced dissociation of the 7SK snRNP complex coincides with enhanced Tat-dependent P-TEFb recruitment to the HIV LTR and the stimulation of proviral transcription elongation [53]. These effects are extremely rapid; within 30 min of engagement of the TCR with α-CD3 and α-CD28 antibodies, or exposure of cells to the protein kinase C (PKC) agonist, phorbol 12-myristate 13-acetate (PMA), 7SK snRNP complex dissociation was induced as measured by gel filtration chromatography or density gradient centrifugation [53], [78]. Both 7SK snRNP complex disruption and HIV transcription elongation could be partially blocked by U0126, an inhibitor of the MAPK/ERK pathway, suggesting that these kinases participate in the signaling cascade leading to P-TEFb activation [53]. To determine whether post-translational modifications are associated with the dissociation of P-TEFb from 7SK snRNP and its enhanced interaction with Tat we used an affinity purification–tandem mass spectrometry approach.

**Figure 1:**
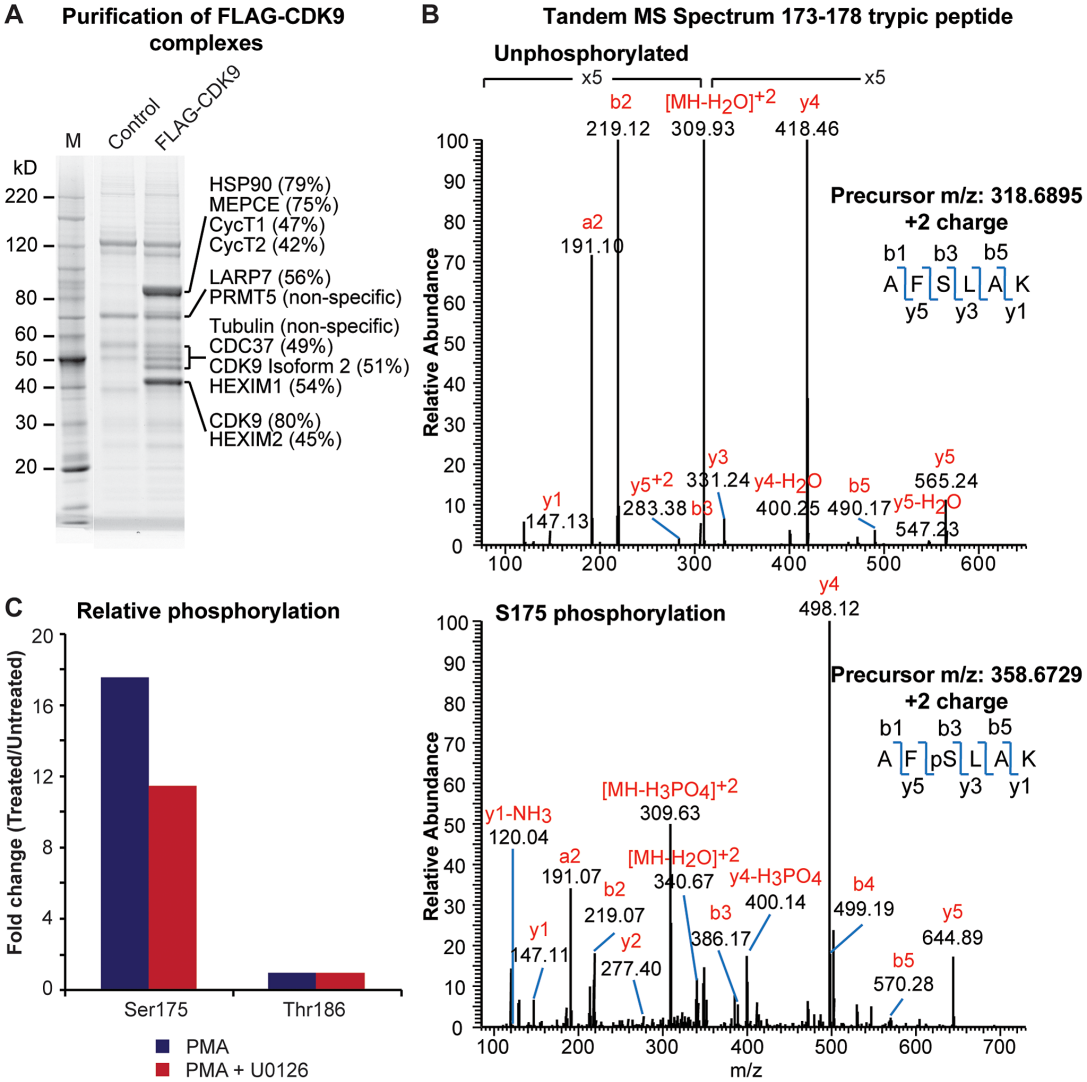
Ser175 phosphorylation of CDK9 is rapidly induced by T-cell activation signals. (A) Affinity purification of FLAG-CDK9 complexes from Jurkat 2D10 cells and their identification by mass spectrometry. The percent values indicate the sequence coverage of the identified proteins. (B) Manually annotated MS/MS fragmentation spectra of the unmodified (upper) and phosphorylated (lower) CDK9 AFSLAK tryptic precursor peptides. (C) Ratio of phosphorylation of CDK9 at Ser175 and Thr186 in PMA stimulated (50 ng/ml) versus untreated cells with or without pretreatment with 20 µM U0126.

As shown in **Fig. 1A**, P-TEFb complexes were isolated from Jurkat T-cells expressing FLAG-CDK9 isoform 1 from a MSCV-based retroviral vector both before and shortly after stimulation with PMA. Gel electrophoresis of the purified complexes revealed a high degree of homogeneity in the samples, with several of the most abundant bands corresponding to CDK9, CycT1 and HEXIM1. Analysis of the individual bands by a nano LC-MS/MS method identified all known 7SK snRNP protein components each with sequence coverage between 42% and 80% of the proteins (**Fig. 1A**). As shown in **Tables 1** **to** **5**, CycT1, CDK9 isoforms 1 and 2, HEXIM1 and HEXIM2 each carry extensive known and novel post-translational modifications (PTMs) including numerous phosphorylation, acetylation and methylation sites that have not been previously identified.

**Table 1:** Post-translational modifications (PTMs) of CDK9 Isoform 1 identified by tandem mass spectrometry analysis.

PTM Type	PTM ID	Modified Peptide	Conditions Detected	Fold change (PMA)	Fold change (TCR)	PeptideScore/Expected Score
Phosphorylation	Ser-7	4–21	TCR			23/0.04
Phosphorylation	Ser-175	173–178	All	17.54× Increase	1.96× Increase	41/0.0013
Phosphorylation	Thr-186	179–188	All	No Change	No Change	32/0.013
Phosphorylation	Ser-317	304–325	PMA			22/0.04
Phosphorylation	Tyr-338	326–343	TCR			30/0.05
Methylation	Lys-21	4–21	PMA, TCR	No Change	2.66× Increase	28/0.028
Methylation	Lys-35	25–35	No Stim. TCR		2.64× Increase	23/0.047
Acetylation	Lys-35	25–37	All	No Change	2.33× Increase	62/0.00007
Acetylation	Lys-56	50–65	All	2.21× Increase	No Change	35/0.020
Acetylation	Lys-68	66–74	No Stim. PMA	6.90× Decrease		35/0.018
Acetylation	Lys-74	69–74	No Stim.			34/0.017
Methylation	Arg-86	75–86	All	No Change	No Change	59/0.00027
Methylation	Lys-127	121–128	PMA, TCR	12.35× Increase	40.89× Increase	42/0.0091
Acetylation	Lys-127	121–128	PMA, TCR	36.51× Increase	34.45× Increase	46/0.005
Acetylation	Lys-164	160–172	All	No Change	No Change	73/0.0001
Acetylation	Lys-178	173–184	All	No Change	No Change	61/0.00023
Acetylation	Lys-269	265–272	All	No Change	No Change	35/0.047
Acetylation	Lys-345	345–358	No Stim. PMA	8.5× Decrease		69/0.00005
Methylation	Arg-370	359–370	All	1.52× Increase	1.57× Increase	55/0.0004

Fold-changes in PTM levels after PMA or TCR activation are relative to the non-stimulated condition. Analyses of the CDK9 isoform 1 was performed using mass spectrometry data from FLAG-CDK9 affinity isolates.

**Table 2:** Post-translational modifications (PTMs) of CDK9 Isoform 2 (117 amino acid extension at N-Terminus) identified by tandem mass spectrometry analysis.

PTM Type	PTM ID	Modified Peptide	Conditions Detected	Fold change (PMA)	Fold change (TCR)	PeptideScore/Expected Score
Phosphorylation	Ser-56	16–58	No Stim. PMA	No Change		35/0.003
Methylation	Arg-8	4–15	No Stim. PMA	4.96× Decrease		28/0.04
Dimethylation	Arg-8	4–15	No Stim. PMA	2.02× Decrease		55/0.0001

Fold-changes in PTM levels after PMA or TCR activation are relative to the non-stimulated condition. Analyses of the CDK9 isoform 2 was performed using mass spectrometry data from FLAG-CDK9 affinity isolates.

**Table 3:** Post-translational modifications (PTMs) of CycT1 identified by tandem mass spectrometry analysis.

PTM Type	PTM ID	Modified Peptide	Conditions Detected	Fold change (PMA)	Fold change (TCR)	PeptideScore/Expected Score
Phosphorylation	Thr-110	107–122	No Stim. TCR		No Change	45/0.003
Phosphorylation	Ser-629	597–631	No Stim. PMA	No Change		32/0.007
Methylation	Lys-376	351–376	No Stim. PMA	No Change		21/0.05
Acetylation	Lys-380	377–386	No Stim. TCR		1.76× Decrease	33/0.04
Acetylation	Lys-386	377–386	No Stim. PMA	1.89× Increase		28/0.045
Acetylation	Lys-492	486–502	All	27.96× Decrease	1.86× Increase	81/0.000005
Acetylation	Lys-707	702–726	No Stim. PMA	3.86× Decrease		27/0.045

Fold-changes in PTM levels after PMA or TCR activation are relative to the non-stimulated condition. Analyses of CycT1 was performed using mass spectrometry data from FLAG-CDK9 affinity isolates.

**Table 4:** Post-translational modifications (PTMs) of HEXIM1 identified by tandem mass spectrometry analysis.

PTM Type	PTM ID	Modified Peptide	Conditions Detected	Fold change (PMA)	Fold change (TCR)	PeptideScore/Expected Score
Phosphorylation	Thr-270	266–284	No Stim.			46/0.05
Phosphorylation	Tyr-271	266–284	PMA			42/0.042
Phosphorylation	Tyr-274	266–284	PMA			37/0.05
Phosphorylation	Ser-355	352–359	No Stim.			54/0.00015
Phosphorylation	Ser-237	233–265	No Stim. PMA	No Change		84/0.0000007
Phosphorylation	Ser-233, Thr-236	233–265	PMA			59,0.000064
Phosphorylation	Ser-233, Ser-237	233–265	PMA			44,0.0017
Phosphorylation	Ser-233, Ser-252	233–365	No Stim.			42/0.0029
Methylation	Arg-146	130–146	PMA			42/0.003
Methylation	Arg-273	266–273	PMA			36/0.05
Acetylation	Lys-284	274–284	No Stim. PMA	2.06× Increase		38/0.04
Acetylation	Lys-289	285–296	No Stim.			46/0.006
Methylation	Lys-296	284–296	No Stim.			41/0.015
Methylation	Arg-347	334–347	No Stim.			32/0.05

Fold-changes in PTM levels after PMA or TCR activation are relative to the non-stimulated condition. Analyses of the HEXIM1 PTMs were performed using data from both FLAG-CDK9 and FLAG-HEXIM1 affinity isolates.

**Table 5:** Post-translational modifications (PTMs) of HEXIM1 identified by tandem mass spectrometry analysis.

PTM Type	PTM ID	Modified Peptide	Conditions Detected	Fold change (PMA)	Fold change (TCR)	PeptideScore/Expected Score
Phosphorylation	Ser-29	23–36	All	2.95× Decrease	3.26× Decrease	39/0.0047
Phosphorylation	Thr-32	23–36	No Stim. TCR		3.26× Decrease	34/0.015
Phosphorylation	Ser-29, Thr-32	23–36	All	1.67× Decrease	1.87× Decrease	55/0.00011
Phosphorylation	Ser-29, Thr-32, Ser-39	23–48	All	No Change	No Change	62/0.000055
Phosphorylation	Thr-46	37–48	No Stim. PMA	No Change		34/0.015
Phosphorylation	Ser-76	74–88	No Stim. PMA	No Change		74/0.000006
Methylation	Arg-199	192–199	No Stim			34/0.05
Dimethylation	Arg-215	211–222	No Stim			37/0.047
Dimethylation	Arg-222	211–222	No Stim			39/0.025

Fold-changes in PTM levels after PMA or TCR activation are relative to the non-stimulated condition. Analyses of the HEXIM2 PTMs were performed using data from FLAG-CDK9 affinity isolates.

**Figure 2:**
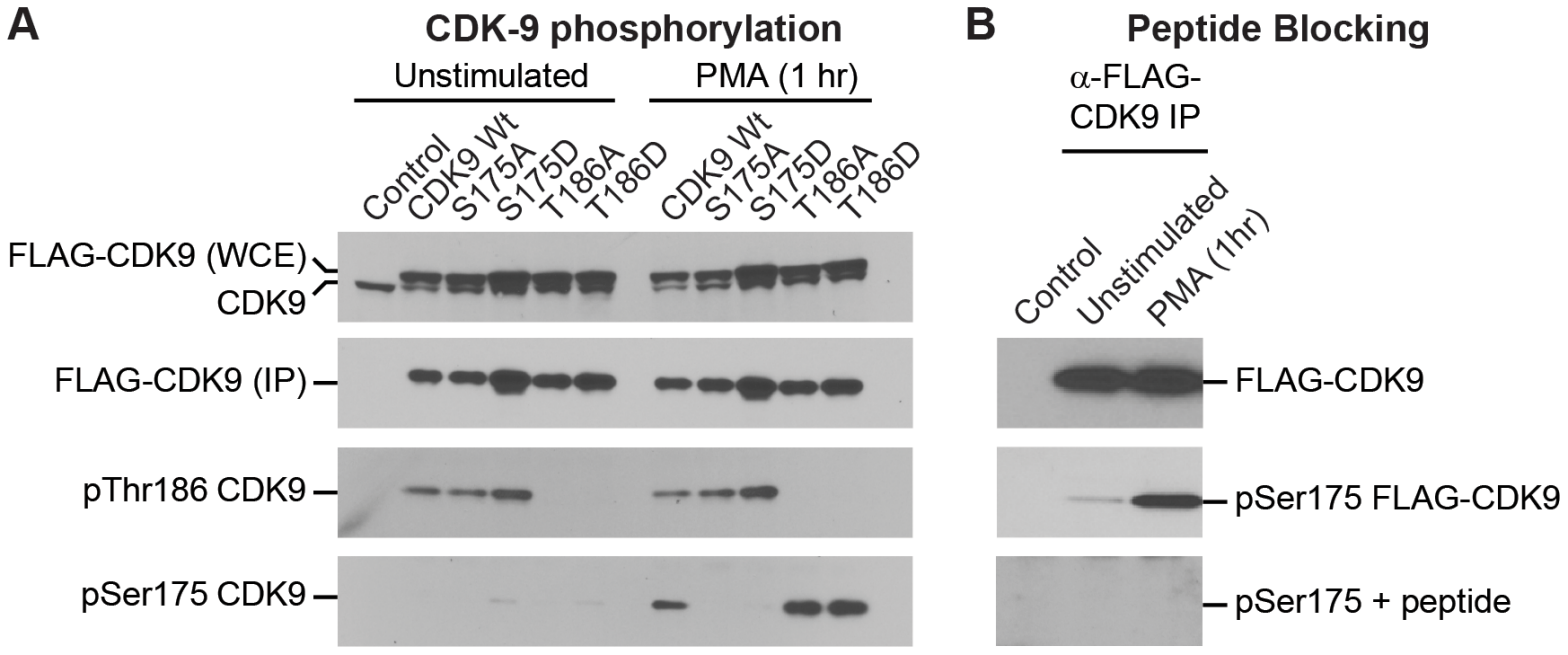
Signal-dependent phosphorylation of CDK9 at Ser175. (A) Detection of Ser175 phosphorylation by Western blotting after 1 h PMA (50 ng/mL) stimulation for wild type CDK9 and the T186A and T186D mutants. FLAG-CDK9 carrying the wild type sequence, or the S175A, S175D, T186A, or T186D mutations was stably expressed in latently infected Jurkat 2D120 cells using the MSCV retroviral expression system. Top panel: Whole cell extracts used for immunoprecipitation were immunoblotted for total CDK9. Note the slower migration of the ectopically expressed FLAG-CDK9 compared to the endogenous CDK9. Bottom three panels: Anti-FLAG-CDK9 immunoprecipitates were screened by immunoblotting for CDK9, pThr186, and pSer175 using a polyclonal antibody derived using a 19-residue peptide carrying a pSer175 epitope. (B) Validation of the epitope specificity of the pSer175 CDK9 antibody by peptide blocking. Purified antibody was pre-incubated overnight with pSer175 peptide epitope prior to immunoblotting anti-FLAG-CDK9 immunoprecipitates derived from control Jurkat T-cells, or 2D10 cells expressing FLAG-CDK9 before and after stimulation for 1 hr by PMA.

The CDK9 isoform 1 (**Table 1**) was consistently identified with >75% sequence coverage that allowed for a near-complete mapping of its PTMs under both basal and activated conditions (**Fig. S1** provides a representative listing of the identified peptides from CDK9 and a Mascot protein database search/analysis). The most pronounced increase in the phosphorylation of CDK9 following PMA treatment of cells was phosphorylation of Ser175 (pSer175), a highly conserved residue located in the activation loop of the kinase (**Fig. 2**). Quantitative MS data from a representative experiment are shown in **Fig. 1C** and **Fig. S2**. Phosphorylation in three separate experiments was enhanced 14.3±1.7 fold over basal levels after 1 h PMA stimulation. U0126, partially blocked, but did not eliminate pSer175 formation, suggesting that this modification is not directly the result of ERK kinase activity. Similar results were obtained following TCR stimulation of cells which led to a 2-fold increase in pSer175 levels (**Table 1**). While this work was in progress, Ammosova et al. [79] also identified Ser175 as an *in vivo* phosphorylation site. Their identification was based on Hunter peptide mapping of *in vivo* -labeled ^32^P-peptides followed by confirmation of the peptide sequence using mass spectroscopy.

A second major modification of CDK9 was the acetylation and methylation of Lys127 (**Table 1**). This residue is located on an external surface of CDK9 away from the T-loop and CycT1 interaction interface.

In contrast to phosphorylation at Ser175, the phosphorylation of the activation loop residue Thr186 (pThr186), which is essential for CDK9 enzymatic activity [36], [37], [73], did not change significantly after PMA stimulation. Moreover, while both the modified and unmodified precursor peptides for Ser175 could be readily identified by MS/MS (**Fig. 1B**), only the pThr186 precursor peptide could be identified under basal and stimulated conditions. These observations are consistent with published reports demonstrating that in cells with steady state levels of P-TEFb, CDK9 is constitutively phosphorylated at Thr186 and that pThr186 is not only important for promoting P-TEFb kinase activity but it is also required for P-TEFb to be sequestered into the 7SK snRNP complex [36], [37], [73].

In order to obtain unambiguous, high resolution MS/MS data as cross-verification of the Ser175 phosphorylation event detected in the Velos ion trap (IT) detector, commercially synthesized AFSLAK peptides with and without phosphorylation at Ser175 (ThermoFisher Scientific) were used as controls and subjected to high resolution CID MS/MS data collection (**Fig. S3**). The data showed that the major daughter ions observed in the ion trap (IT) detector (**Fig. 1B**) can be detected with higher accuracy in the FT detector. Due to the much higher precursor ion abundance obtained from the synthetic peptide samples, some additional fragment masses that were undetected in the IT experiment were also seen in the FT MS/MS spectra and provided further verification for the tandem MS-based detection and confirmation of the pSer175 modification.

Virtually all the modifications we found on the other 7SK RNP components (CDK9 isoform 2 (Table 2), CycT1 (Table 3), HEXIM1 (Table 4) and HEXIM2 (Table 5)) have not been previously described. An important exception is that in agreement with Cho et al. [81] we have found acetylation of CycT1 on K386, a region of the molecule that is predicted to form a coiled-coil structure. Acetylation of CycT1 has been associated with shifting the the balance between Hexim1-bound (inactive) and Hexim1-free (active) P-TEFb [81].

It should be noted that the identified PTMs in **Table 1** are based on data obtained in Jurkat T-cells which have assembled 7SK RNP complexes. Primary resting memory CD4+ T-cells are severely restricted in CycT1 and therefore have only miminal levels of 7SK RNP complexes, it is therefore expected that many of these reported modifications are no found in resting memory T-cells, as is the case for pThr186. We therefore expect that the majority of the modifications detected in Jurkat T-cells will only be present in activated T-cells.

### Antibody detection of Ser175 phosphorylation

In order to facilitate the detection of CDK9 Ser175 phosphorylation, we developed a rabbit polyclonal antibody against a peptide containing pSer175. The crude antiserum was initially affinity purified using the phospho-serine peptide and the eluted antibody fractions were further purified by multiple rounds of negative selection with affinity resin for the corresponding unmodified peptide.

Wildtype CDK9 and a series of mutations in Ser175 and Thr186 were stably expressed as FLAG-tagged proteins in Jurkat cells using the MSCV-retroviral expression vector. As shown in **Fig. 2A**, when whole cell extracts from these cells were blotted using α-CDK9 antibody, both the endogenous CDK9 and the FLAG-tagged proteins, which migrate slightly slower than the native CDK9, were detected. Ectopic expression of the FLAG-tagged CDK9 protein reduced endogenous CDK9 expression by approximately 2-fold and resulted in a ratio of FLAG-CDK9 to CDK9 expression of between 2.0 and 3.0. Consequently, the majority of the CDK9 in cells can be recovered in the FLAG-immunoprecipitates.

The FLAG-tagged proteins were immunoprecipitated with α-FLAG antibody and then blotted using α-CDK9, α-pThr186 and α-pSer175 CDK9 antibodies. The blots using the α-pSer175 CDK9 antibody showed a 10.86-,27.49-, and 111.6-fold increase in pSer175 levels following 1 hr PMA stimulation in three different experiments (49.98±31.2) (**Fig. 2A**), consistent with the increases observed in the MS/MS experiments (**Fig. 1**). Further evidence that the antibody we developed is highly selective for pSer175, comes from its failure to react against CDK9 carrying the S175A or S175D mutations after 1 hr PMA stimulation. As an additional control, peptide blocking experiments performed using the phosphorylated peptide showed complete inhibition of antibody binding to the phosphorylated form of CDK9 (**Fig. 2B**).

**Figure 3:**
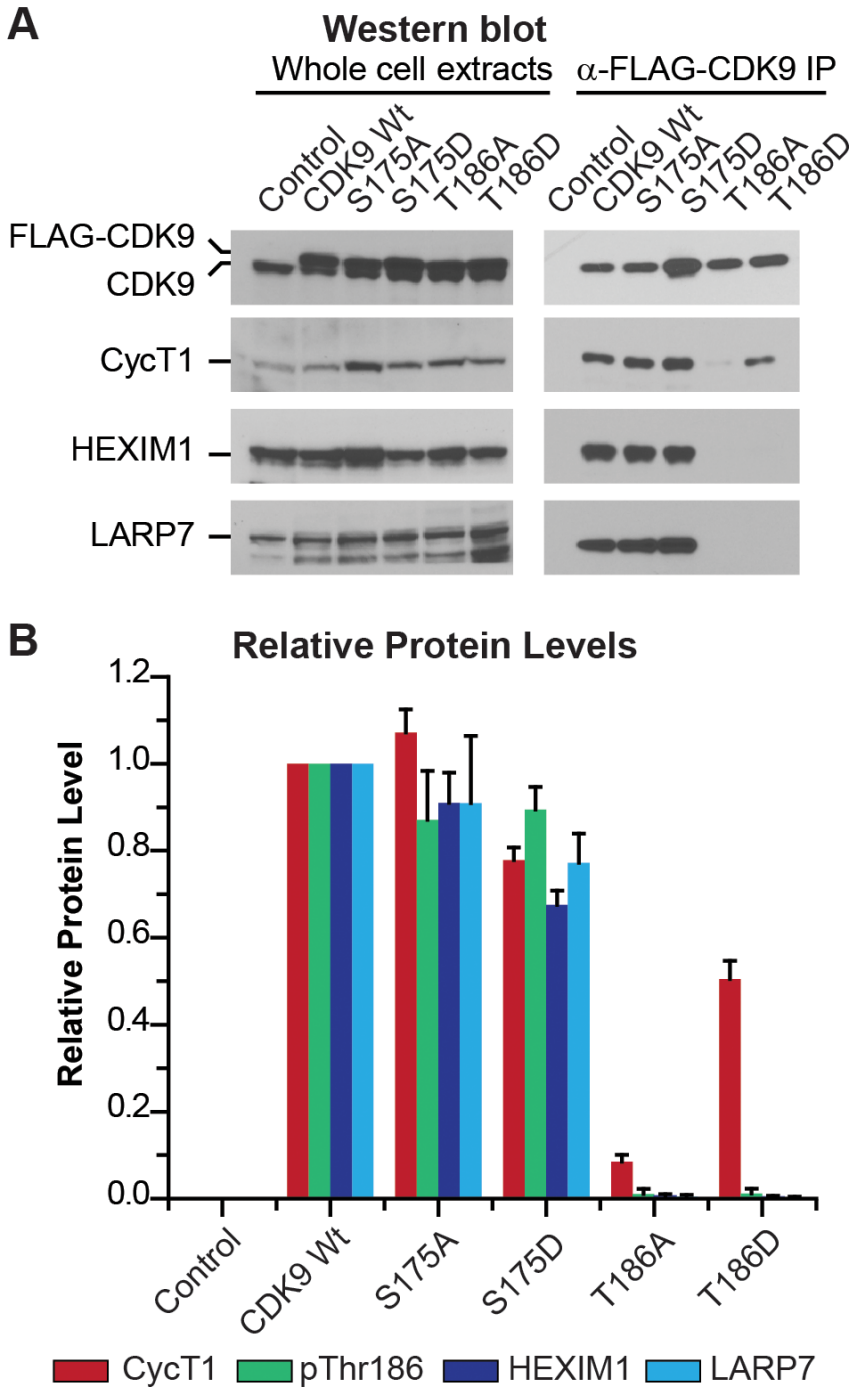
Ser175 is not required for P-TEFb formation and the assembly of the 7SK snRNP complex. (A) FLAG-CDK9 wildtype, S175A, S175D, T186A, or T186D were stably expressed in Jurkat 2D10 cells using the MSCV retroviral expression system. Western blotting analysis was performed on the whole cell extracts (left panels) and the corresponding anti-FLAG-CDK9 immunoprecipitates (right panels) using antibodies against CycT1, CDK9 and the 7SK RNP protein components HEXIM1 and LARP7. (B) Quantitative analysis of protein levels. Protein concentrations were estimated by densitometry of the Western blots and normalized against total immunoprecipitated CDK9. The data are from five independent experiments. Error bars: ± standard error of the mean.

To evaluate the impact of mutations in the T-loop on the assembly of the 7SK snRNP complex we performed Western blotting experiments using 7SK snRNP complexes recovered by affinity chromatography of FLAG-tagged CDK9. As shown in **Fig. 3**, the S175A and S175D mutations of CDK9 did not affect the assembly of the 7SK snRNP complex and permitted efficient co-purification of CDK9, CycT1 and the 7SK snRNP components HEXIM1 and LARP7. For the S175A mutation each of the components was immunoprecipitated with >90% efficiency. Compared to the wildtype CDK9 and the S175A mutant, co-IP of S175D showed somewhat reduced levels of the 7SK RNP complex components (e.g. 77.8%±2.9% CycT1, 67.5%±3.3% HEXIM1 and 77.2%±6.6% LARP7). However, we believe that the association of S175D with the 7SK complex is equivalent to the wildtype since this mutant was expressed at 1.5 to 2.0-fold higher levels than the wildtype CDK9 and the excess CDK9 may be found in association with chaperone proteins [82].

The CDK9 in these complexes was phosphorylated on T186, as demonstrated by Western blotting using a pT186-specific antibody [36] (**Fig. 2A**). Phosphorylation of CDK9 at Thr186 is not only essential for enabling its kinase activity but is also required for CDK9/hCycT1 P-TEFb to become assembled into the catalytically inactive 7SK snRNP [36], [37], [69], [73]. As expected, mutations in Thr186 prevented assembly into the 7SK snRNP complex and therefore co-precipitation of HEXIM1 and LARP7 was reduced by over 99% (**Fig. 3A**). The mutations in Thr186 limited, but did not abolish, CDK9 binding to CycT1. The T186A mutation strongly inhibited CDK9 binding to CycT1 (8.5±1.6% of the wildtype), whereas the T186D mutation only reduced binding to CycT1 to 50.5 5±4.2% (**Fig. 3B**).

Our observation that T186A fails to bind to CycT1 disagrees with previous observations by Yang et al. [10] and Li et al. [9]. A possible molecular explanation for why mutation of Thr186 compromises CDK9 interaction with Cyclin T1 is based on the study by Russo et al. [71] of who clearly demonstrated that in the analgous CDK2-Cyclin A structure the phosphate moiety at the highly conserved CDK T-loop T186 residue serves as an organizing center to coordinate a network of intramolecular and intermolecular hydrogen bonding interactions that stabilize heterodimerization with the Cyclin T1 subunit.

In summary, the preceding immunoprecipitation experiments demonstrate unequivocally that the phosphorylation of CDK9 at Ser175 is not required for the assembly of P-TEFb/7SK snRNP.

### CDK9 phosphorylated at Ser175 is excluded from the 7SK snRNP complex

**Figure 4:**
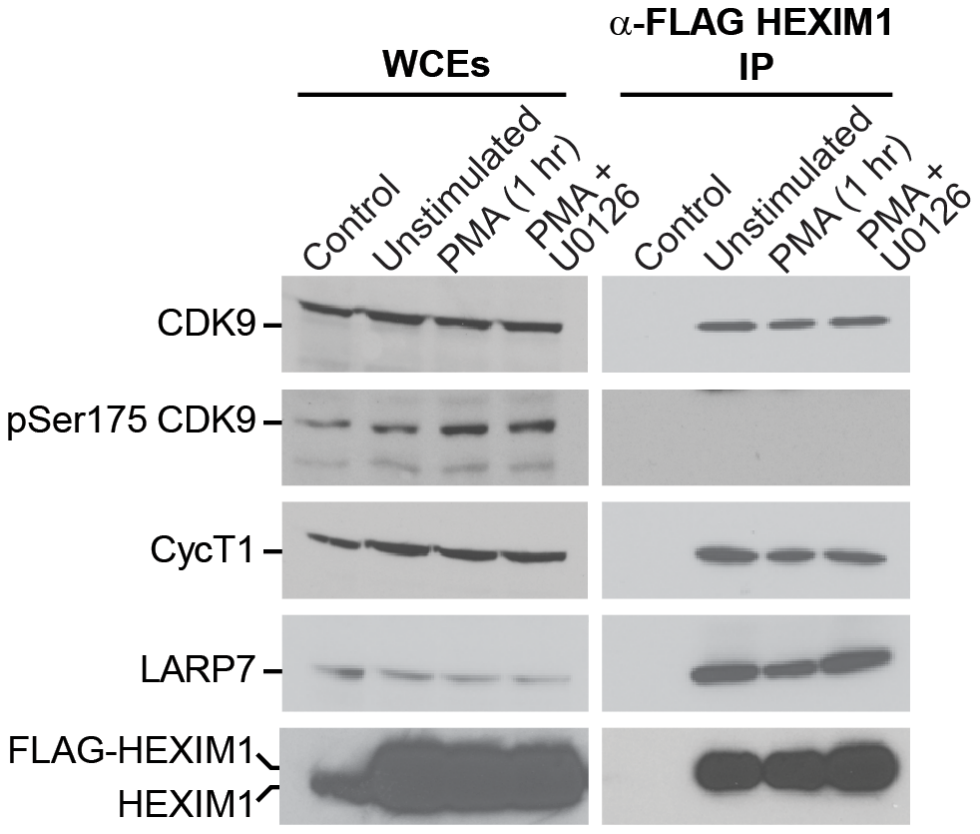
CDK9 phosphorylated at Ser175 is excluded from the 7SK snRNP complex. 2D10 cells were engineered to stably express FLAG-HEXIM1. Left panels: WCEs were prepared from cells that were unstimulated or treated with PMA for 1 h with or without the inhibitor U0126. Right panels: FLAG-HEXIM1 complexes were isolated by anti-FLAG IP followed by elution with FLAG peptide. Immunoblotting was performed on whole cell extracts (input samples) and anti-FLAG immunoprecipitates using antibodies towards CycT1, CDK9, HEXIM1, LARP7, pT186 CDK9, and pSer175 CDK9. The result shown is representative of two different experiments. Note that HEXIM1- P-TEFb found in the HEXIM-associated complexes is devoid of phosphorylation at Ser175 whereas the modification is readily detected in WCEs and in FLAG-CDK9 immunoprecipates (**Fig. 3**).

To determine whether CDK9 can be phosphorylated at Ser175 while assembled within 7SK snRNP, we also carried out an affinity isolation of the 7SK complex from Jurkat T-cells stably expressing FLAG HEXIM1 (**Fig. 4**). In contrast to FLAG-CDK9, FLAG-HEXIM1 is expressed at approximately 3.5-fold higher levels than the endogenous protein in these cell lines. The 7SK RNP complex components (CDK9, CycT1 and LARP7) that co-purified with HEXIM1 under these conditions were analyzed by Western blotting before and after treatment of the cells with PMA. Our previous gel filtration chromatography studies demonstrated that activation of MAPK/ERK by brief stimulation of Jurkat T-cells with PMA induces partial 7SK complex dissociation and release of P-TEFb [53]. Since HEXIM1 only associates with P-TEFb as part of the 7SK complex, any CDK9 that precipitates with HEXIM1 is present in the 7SK complex, while CDK9 that does co-precipitate represents released P-TEFb. As shown in **Fig. 4**, PMA stimulation for 1 h reduced HEXIM1 association with CDK9, Cyclin T1, and LARP7 by 30%, 31%, and 23% respectively, indicating that approximately 30% of the 7SK complexes were disrupted. While we could easily detect endogenous CDK9 in the HEXIM1 immunoprecipitates, it is important to note that pSer175 could not be detected within these complexes even after PMA stimulation when pSer175 levels are readily detected in the whole cell extracts (**Fig. 4**). Thus, CDK9 carrying pSer175 appears to be excluded from the 7SK snRNP complex, and is likely to represent a transcriptionally active form of the P-TEFb enzyme.

### Ser175 regulates Tat and BRD4 binding to P-TEFb

**Figure 5:**
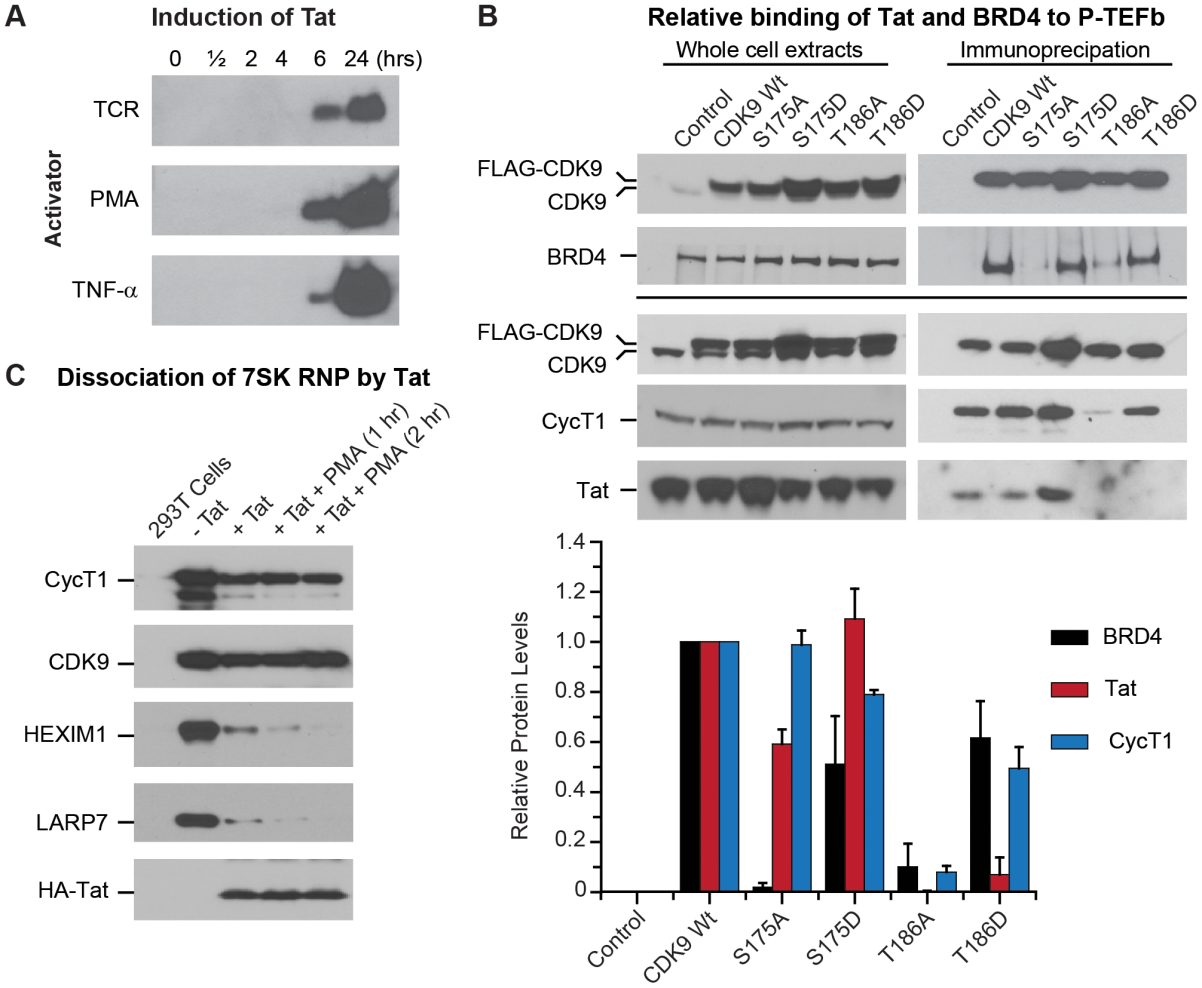
Ser175 mediates the binding of Tat to CDK9 and BRD4. (A) Induction of Tat expression in latently infected Jurkat 2D10 cells. WCEs were prepared from 2D10 cells and treated for the indicated times with PMA (50 ng/ml), TNF-α (10 ng/ml), or a combination of anti-CD3 (0.125 µg/ml) and anti-CD28 (1 µg/ml) mAbs. The extracts were then subjected to Western blotting using a Tat monoclonal antibody.(B) Relative binding of Tat and BRD4 to P-TEFb. Top: Western blots of WCEs (left panels) or FLAG-CDK9 immunoprecipitates (right panels). Top two panels show an experiment detecting BRD4 association with FLAG-CDK9 while the bottom three panels show a separate experiment detecting CycT1 and Tat association with FLAG-CDK9. Graph shows the relative levels of co-precipitated BRD4, Tat, and CycT1 normalized to corresponding total CDK9 levels. Data are from three different experiments. Error bars: ± standard error of the mean. (C) Tat-dependent and signal-dependent dissociation of P-TEFb from 7SK snRNP. 293T cells stably expressing FLAG-tagged CDK9 were transiently transfected with HA-tagged Tat. Immunoprecipitation was performed using anti-FLAG antibody followed by immunoblotting using antibodies against CycT1, CDK9, HEXIM1, LARP7, and Tat.

As shown in **Fig. 5A**, Jurkat T-cells latently infected with HIV express subthreshold levels of Tat that are undetectable by Western blotting [53]. Following stimulation of the cells by α-CD3 and α-CD28 antibodies, PMA, or TNF-α, Tat levels progressively increase reaching maximum levels by 24 hrs.

We took advantage of this induction system to examine the extent to which the phosphorylation of CDK9 at Ser175 may alter the affinity of P-TEFb for Tat and BRD4. Latently infected Jurkat T-cells engineered to stably express FLAG-tagged versions of CDK9 wildtype, S175A, S175D, T186A, or T186D *in trans* were induced by 16 hr treatment with TNF-α prior to the affinity isolation of P-TEFb complexes by anti-FLAG IP (**Fig. 5B**). After normalizing to the corresponding CDK9 levels, 59.2±5.9% of Tat remained associated with S175A CDK9 compared to wildtype (**Fig. 5B**). By contrast the S175D phosphomimetic mutation modestly increased the association of CDK9 with Tat (109.2%±11.9%).

Consistent with the results in **Fig. 3**, both the S175A and S175D mutants bound CycT1 with nearly wildtype efficiency (S175A: 98.9±5.7%; S175D: 78.9±1.9%) (**Fig. 5B**). However, it is important to note that the two mutations had disproportionate effects on BRD4 binding. The S175A mutation significantly reduced BRD4 binding to 1.9±1.7% whereas the S175D mutation bound BRD4 with 51.1±19.3% of the wildtype efficiency. There was no significant difference between the CDK9 association profiles of wildtype Tat and the H13L Tat in these experiments.

As shown in **Fig. 5B**, the T186A mutation severely reduced binding to CycT1 (8.0±2.5%) and consequently abrogated Tat interaction with CDK9 (0.3%±0.3%). The phosphomimetic mutation T186D showed somewhat reduced BRD4 binding (61.6±14.7% of wildtype) and a similarly reduced level of CycT1 binding (49.4±8.5%). However Tat binding to T186D CDK9 was severely restricted (7.0%±7.0%). Thus, both T186 mutations are likely to alter the conformation of the activation loop in ways that interfere with potential intermolecular electrostatic interactions between CycT1, BRD4 and the N-terminus of Tat.

To study the impact of Tat expression on 7SK snRNP complex dissociation, and to evaluate whether Ser175 phosphorylation could enhance Tat-dependent disruption of the 7SK RNP complex, we also employed an immunoprecipitation strategy. For these experiments, 239T cells stably expressing FLAG-tagged CDK9 were transiently transfected with HA-tagged Tat since only trace amounts of HA-Tat could be constitutively expressed in Jurkat cells from retroviral vectors. Phosphorylation of Ser175 was induced by treatment of the cells for 1 hr or 2 hrs with PMA. As shown in **Fig. 5C**, ectopic expression of HA-Tat caused disruption of the 7SK snRNP complex and resulted in release of 71% of the HEXIM1 and 82% of the LARP7 from the affinity purified P-TEFb fractions. Stimulation of cells with PMA increased the efficiency of the disruption of the 7SK snRNP complex by Tat, with the result that after 2 hrs of treatment with PMA of 2.4% of the HEXIM1 and 0.04% of the LARP7 copurified with the P-TEFb complex.

As, expected the HA-Tat copurified with the released P-TEFb. After normalization for CDK9 levels there was a moderate 12% increase in the amount of Tat associated with CDK9 after 1 hr of PMA treatment. These results are consistent with our hypothesis that Ser175 phosphorylation, while not obligatory for Tat binding, can enhance the interactions between P-TEFb and Tat.

### Ser175 phosphorylation is not due to autophosphorylation

Previous reports have suggested that phosphorylation of Ser175 might be due to CDK9 autophosphorylation [79]. Since Thr186 is essential for the kinase activity of CDK9 [66], [69], [71], we decided to test whether T186A and T186D could become phosphorylated at Ser175. In the experiment shown in **Fig. 2A**, Ser175 phosphorylation increased 37- and 32-fold, respectively for the T186A and T186D mutations after 1 hr PMA treatment of cells. Thus, neither the assembly into the 7SK RNP complex, nor the enzymatic activity of CDK9 is required for S175 phosphorylation.

**Figure 6:**
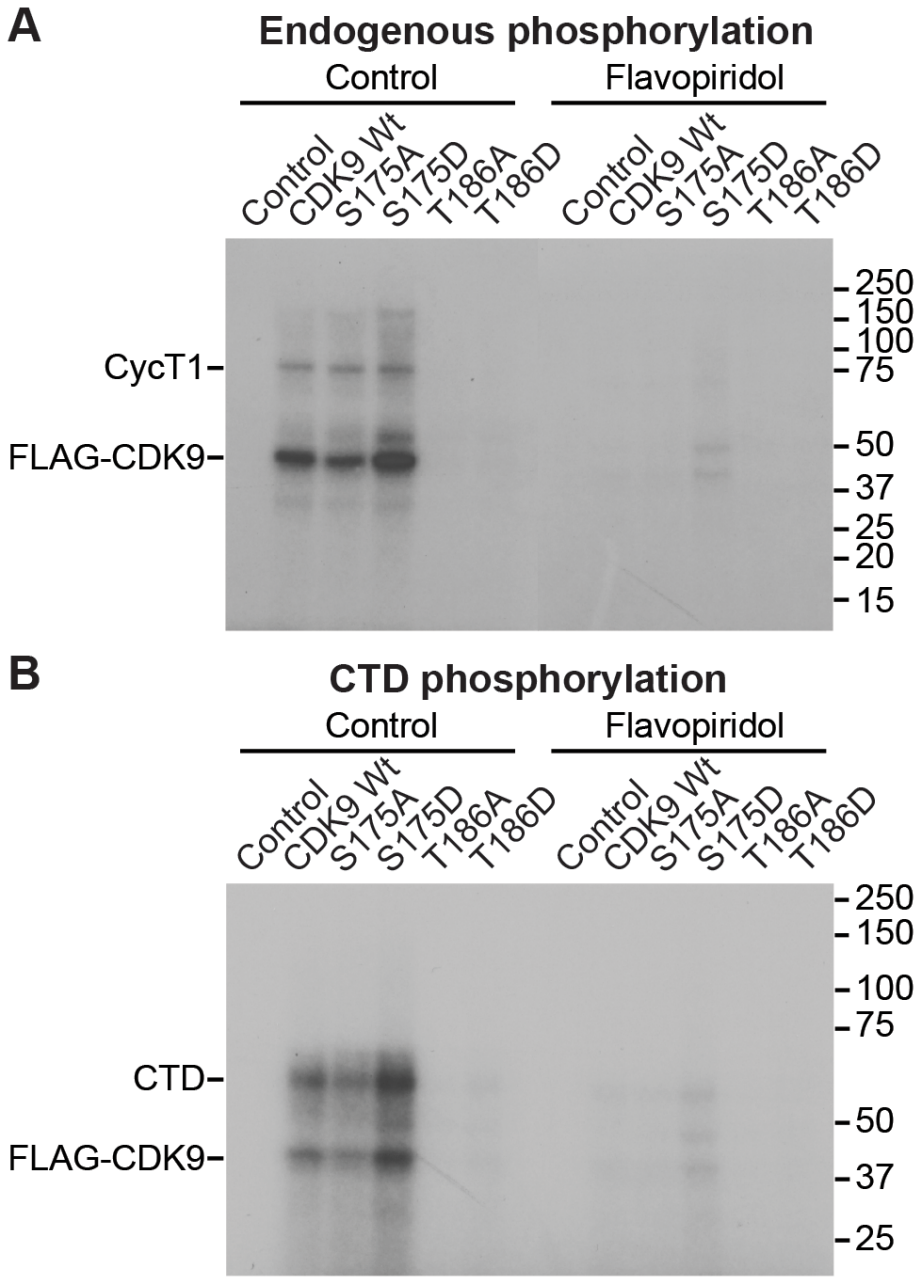
CDK9 kinase activity does not require S175. Radioactive *in vitro* kinase assays perfomed using FLAG-CDK9 complexes isolated from Jurkat 2D10 cells stimulated for 20 h with 10 ng/mL TNF-α to induce Tat expression. Western blots of these IPs and the corresponding WCEs are shown in the lower panel of **Fig. 5B**. The kinase assays were performed in the absence (A) or presence (B) of 250 ng of His-tagged full length human pol II CTD repeat substrate. Inhibition of activity by pretreatment with 100 nM flavopiridol confirms the activity in both assays to be CDK9 kinase. Note that the S175A and S175D mutations display wildtype kinase activity in both assays.

We also performed *in vitro* kinase assays using affinity purified P-TEFb in the absence (**Fig. 6A**) and presence of a RNAP II CTD peptide (**Fig. 6B**). For these experiments, Tat was induced by overnight treatment of latently infected Jurkat cells with TNF-α prior to purification of complexes carrying P-TEFb. Immunoblots of these samples are shown in **Fig. 5B** (lower panel).

P-TEFb carrying wildtype CDK9 showed high enzymatic activity in both assays and was used to normalize the data. The kinase activity was also strongly inhibited by 100 nM flavopiridol, a potent CDK9 inhibitor, demonstrating that it is unlikely to be due to contaminating kinases.

The S175A mutation had wildtype activity (90.1% and 98.0% in the autophosphorylation and CTD kinase assays). By contrast, the S175D phosphomimetic mutation increased CDK9 kinase activity to 117% in the autophosphorylation assay and 158.7% in the CTD assay, consistent with a stabilization of the Tat-P-TEFb interface by this mutation. In partial disagreement with our results, Yang et al. [80] have reported that the Ser175Ala (S175A) mutation inactivates P-TEFb catalytic activity as assessed by *in vitro* kinase assays while the S175D had wildtype activity. Using an *in vitro* transcription assay they were able also to demonstrate that S175D CDK9/hCycT1 can mediate Tat-dependent full length transcription from the HIV LTR [80].

Since the S175A and S175D, sequences cannot be phosphorylated on Ser175, the CDK9 autophosphorylation events detected in these assays are clearly occurring on other sites, most likely in the C-terminal region [83]. As expected the T186A and T186D mutations, which fail to assemble properly with CycT1 severely inhibited P-TEFb kinase activity and reduced kinase activity to less than 2% of the wildtype.

We conclude that the signal-dependent phosphorylation of Ser175 is independent of CDK9 autophosphorylation and can occur only on P-TEFb that has dissociated from the 7SK snRNP complex.

### Transactivation of latent HIV proviruses by P-TEFb carrying mutations in CDK9 Ser175

Since mutations in Ser175 have an impact on the binding of P-TEFb to both Tat and BRD4 we decided to evaluate whether expression *in trans* of CDK9 carrying mutations in Ser175 and Thr186 had an effect on latent HIV proviral expression. For these experiments we compared cells carrying proviruses with H13L Tat (2D10), wildtype Tat (E4) and the inactive C22G Tat (2B2D). The proviruses in these experiments also carried a d2EGFP fluorescent marker permitting measurements of proviral expression by flow cytometry.

**Figure 7:**
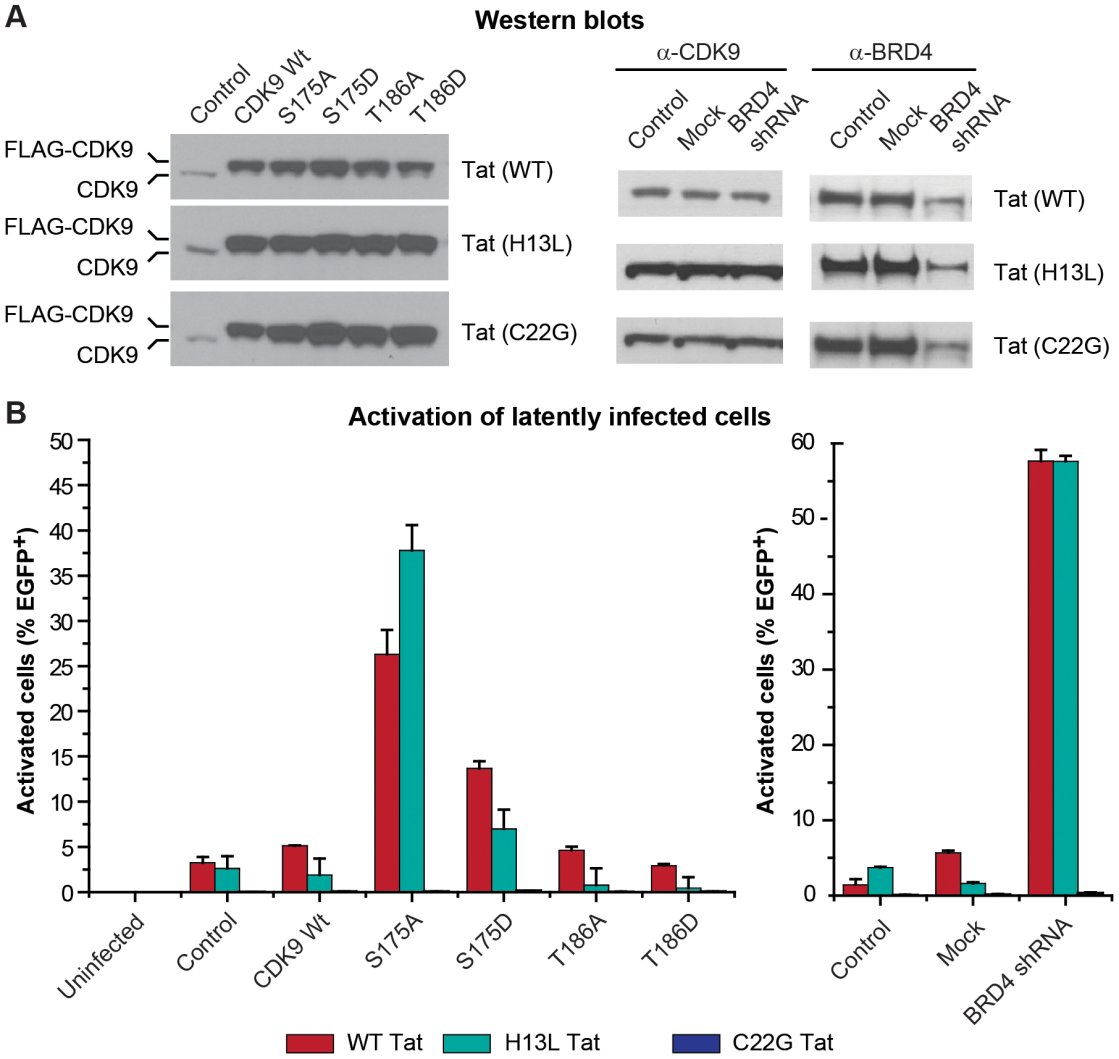
Reactivation of latent HIV proviruses by expression of P-TEFb carrying mutations in CDK9 Ser175 or by knockdown of BRD4 expression. (A) Western blots. Left panels: Stable ectopic expression of FLAG-CDK9 wildtype, S175A, S175D, T186A, or T186D in latently infected E4 (Wildtype Tat), 2D10 (H13L Tat), and 2B2D (C22G Tat) Jurkat T-cells using the MSCV retroviral expression system. Right panels: Stable knockdown of BRD4 expression in E4, 2D10, and 2B2D cells using lentiviral expressing shRNA constructs. (B) Activation of latently infected cells. Left graph: Reactivation of proviral gene expression by S715D CDK9 or S175A CDK9 requires functional Tat. Three weeks post-transduction with FLAG-CDK9 wildtype, S175A, S175D, T186A, or T186D the cells were analyzed by flow cytometry for the spontaneous reactivation of latent proviruses. Right graph: Knockdown of BRD4 reactivates HIV proviral expression in a Tat-dependent manner. Three weeks post-infection with mock or BRD4-specific shRNA lentiviral constructs the cells were analyzed by flow cytometry for the spontaneous reactivation of latent proviruses.

Essentially identical results were obtained with both the H13L Tat and wildtype Tat proviruses. As shown in **Fig. 7A**, the stable expression of S175D CDK9 in latently infected Jurkat E4 cells induced significant basal HIV proviral gene expression (Wildtype CDK9, 5.12±0.06% (E4); S175D 13.66±0.79% (E4)). This is consistent with the idea that introduction of a negative charge at position 175, either through phosphorylation, or because of the phosphomimetic mutation, enhances CDK9 binding to Tat. Similarly, in our earlier study, we showed that both PMA and TCR activation enhanced HIV proviral transcription [53].

We were surprised to observe that expression of the S175A mutation more potently reactivated latent HIV proviral gene expression than the S175D mutation (Wildtype CDK9, 5.12±0.06% (E4); S175A 26.28±2.70% (E4)) (**Fig. 7B**). Ammosova et al. [79], also observed that expression of S175A is able to activate HIV transcription.

Measurable induction of latent proviral gene expression by the S175A and S175D CDK9 mutants required the presence of functional Tat protein. There was no induction of HIV proviral expression above control levels in cells carrying the inactive C22G Tat (**Fig. 7B**).

Since we have observed that S175A somewhat reduces CDK9 association with Tat, and we and Yang et al. [80] have found that this mutation nearly completely abolished its association with BRD4 [80], [84], it seems likely that the proviral reactivation induced by expression of CDK9 carrying the S175A mutation results from altering competition between Tat and BRD4 for P-TEFb. Consistent with this idea, and in agreement with several recent publications [85], [86], [87], the stable knockdown of BRD4 expression by shRNA also led to a robust reactivation of latent proviral gene expression (**Fig. 7B**). Proviral reactivation due to the knockdown of BRD4 expression was Tat-dependent and proviral expression was not detected following BRD4 knockdown in latently infected cells carrying the inactive C22G Tat (**Fig. 7B**).

### Phosphorylation at Ser175 enhances the interactions between Tat with P-TEFb

**Figure 8:**
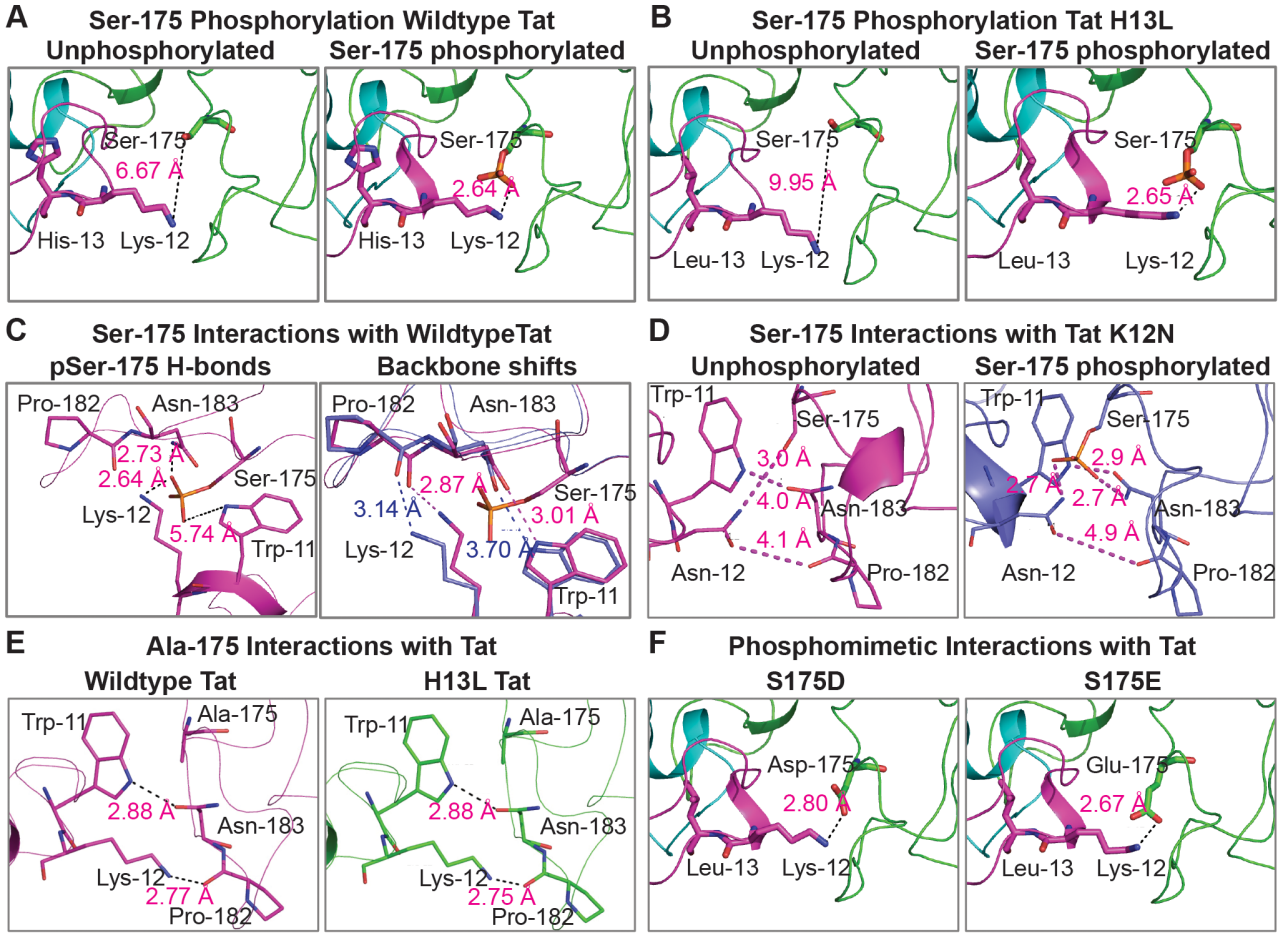
Energy minimized models of the interaction of the N-terminal region of Tat with the activation loop of CDK9. (A) Phosphorylation of Ser175 at the activation loop of CDK9 favors a hydrogen bonding interaction with Lys12 of wild type Tat. (B) pSer175 interactions with Lys12 are unaffected by the H13L mutation. (C) Impact of Ser175 phosphorylation on preexisting intermolecular interactions between CDK9 and Tat. (D) Modeling of K12N Tat/CDK9 interactions with or without the phosphate moiety at Ser175. (E) Tat interactions with CDK9 S175A in the wildtype and H13L backgrounds. (F) Tat interactions with the CDK9 phosphomimetic mutants S175D and S175E.

The recently published Tat/P-TEFb X-ray structure by Tahirov et al. demonstrated that though Tat forms the majority of its contacts with hCycT1, it may also form electrostatic interactions with the activation loop of CDK9 [88]. Two hydrogen bonds are postulated to be formed between Pro182 and Asn183 of CDK9 and Lys12 and Trp11 of Tat, respectively. These Tat/CDK9 contacts are thought to have a role in stabilizing an N-terminal α-helical fold in Tat that contains both Trp11 and Lys12 [88]. Ser175 is unmodified in the X-ray structure and is situated within the activation loop about 6.7 Å away from Lys12 of Tat (**Fig. 8A**).

To examine whether modification of Ser175 could be accommodated in the Tat/P-TEFb structure, and assess whether it can potentially make a contribution to Tat/CDK9 intermolecular interactions we modeled and energy minimized a wide range of sequence variations (**Fig. 8**). The modeling revealed that pSer175 is able to form an intermolecular hydrogen bond with Tat Lys12, bringing the phosphate to 2.6 Å of the amino group of Lys12 (**Fig. 8A**). Similar results were obtained when the modeling was performed using Tat carrying the H13L mutation (**Fig. 8B**). In both cases the phosphate fits nicely into a “pocket” in the structure and can be accommodated with no significant change in the conformation of the CDK9 activation loop backbone (**Fig. 8C**). Lys 12 is present in the consensus clade B sequence. However, Asn12 is the consensus residue in each of the non-clade B Tat sequences [89]. The modeling suggests that Asn12 is also easily accommodated in the structure and is also able to form a hydrogen bond with pSer175 (**Fig. 8D**).

Additional modeling was performed using mutations in Ser175. Modeling of these substitutions in CDK9 suggested that S175A induces a slight change in the Tat backbone structure but does not lead to disruption of the interactions between Tat Trp11 and CDK9 Asn183, or the interactions between Tat Lys12 and CDK9 Pro182 (**Fig. 8E**). Modeling of the phosphomimetic substitutions S175D and S175E suggests that they are both able to form hydrogen bonds with Tat Lys12 (**Fig. 8F**).

In summary, consistent with the experimental data described above, the modeling studies suggest that Ser175 phosphorylation and the S175A and S175D mutations are easily accommodated in the P-TEFb-Tat structure and that S175 phosphorylation potentially stabilizes the interactions between Tat and CDK9.

### P-TEFb carrying pSer175 is found in nuclear speckles

In primary resting memory CD4^+^ T cells the transcriptional activity of CDK9 is restricted due to the absence of its major regulatory partner hCycT1 and lack of Thr186 phosphorylation [36], [37], [38]. The activation of P-TEFb in resting memory CD4^+^ T cells may therefore require multiple sequential events that involve induction of hCycT1 expression, formation of P-TEFb and Thr186 phosphorylation of CDK9, initial assembly of P-TEFb into 7SK snRNP, and release of P-TEFb from the inactive 7SK complex and its eventual mobilization toward RNAP II transcribed genes (see **Discussion**).

**Figure 9:**
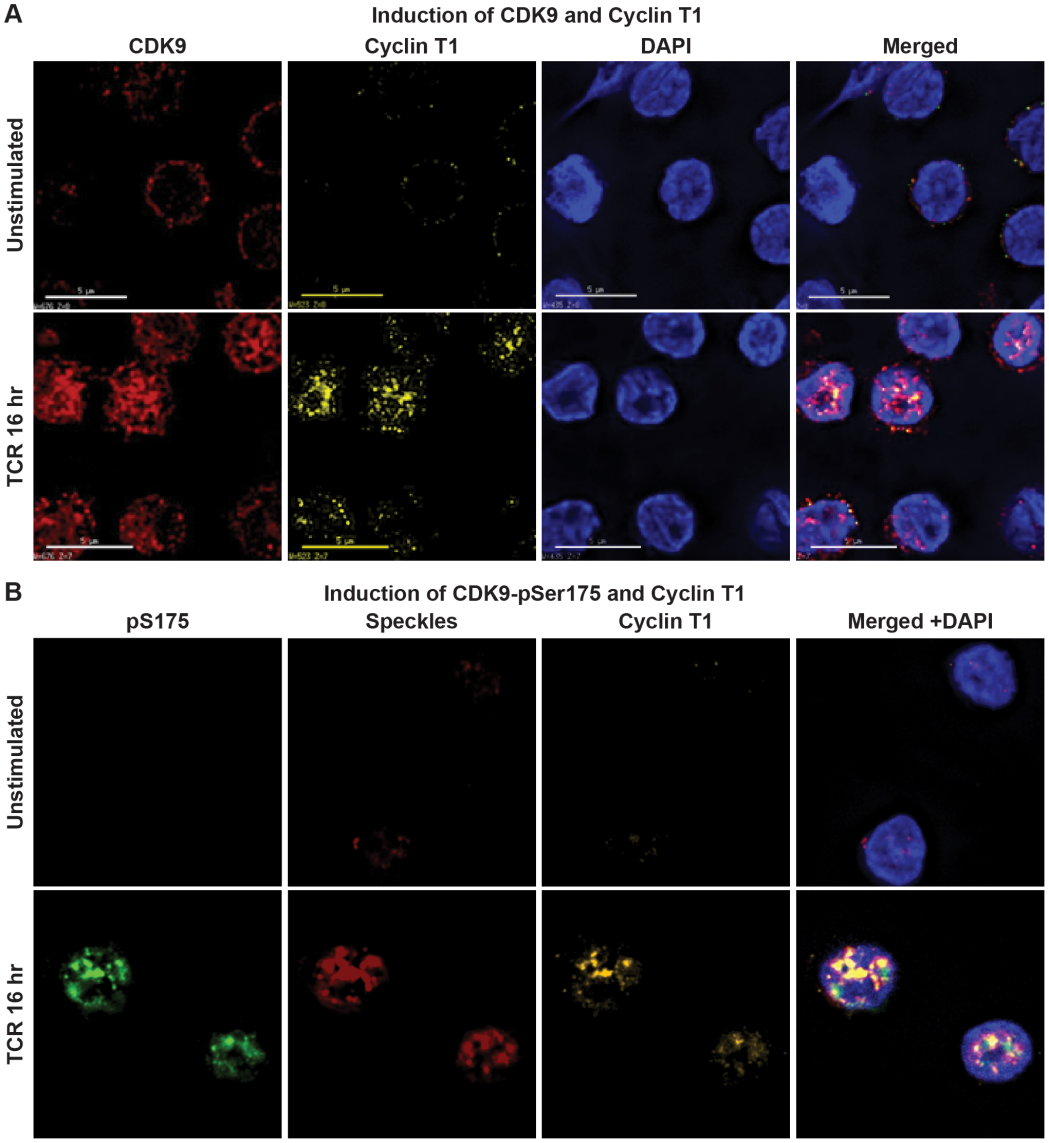
Induction of CycT1 expression and CDK9 Ser175 phosphorylation after activation of primary memory CD4^+^ T-cells via the T-cell receptor. (A) Induction of CDK9 and Cyclin T1. Memory CD4^+^ T cells isolated from a healthy donor were stimulated for 16 hr with α-CD3 and α-CD28 mAbs to activate the TCR, and immunostained with flourophore-conjugated antibodies against CDK9 (red), CycT1 (yellow). Nuclear DNA was stained with DAPI (blue). (B) Cells stained for pSer175 CDK9 (green), nuclear speckles (red), CycT1 (yellow) and nuclear DNA (blue). Images were obtained using a DeltaVision deconvolution microscope.

Support for this model comes from studies of the subcellular localization of hCycT1, pSer175 CDK9, and pThr186 CDK9 in resting and activated CD4^+^ T cells by immunofluorescence microscopy (**Fig. 9**). In unstimulated resting memory T-cells hCycT1 levels are severely restricted and CDK9 is largely restricted to the cytoplasm (**Fig. 9A**). and pSer175 levels are undetectable (**Fig. 9B**).

Upon activation of the cells through the TCR there is upregulation of hCycT1 and concomitant increases in CDK9 and pSer175 CDK9 levels. In a recent publication, Budhiraja et al. [72] also observed upregulation of CDK9 and pThr186 upon activation of resting T-cells. We found that after TCR activation, hCycT1, pSer175 CDK9, colocalize with each other and with a marker of nuclear speckles and exhibited a punctate nucleoplasmic staining (**Fig. 9B**). These observations are consistent with those of Dow et al. [73] who have proposed that nuclear speckles are regions of active cellular gene transcription and sites of P-TEFb/7SK snRNP localization. The confinement of P-TEFb within nuclear speckles would therefore allow activated P-TEFb to be conveniently transferred from the 7SK RNP complex to transcribed genes in response to the appropriate extracellular signals of T cell activation. As shown in **Fig. S4A**, the immunofluorescent signal to pS175 is specific for this modified form of CDK9 since it can be effectively blocked by the pS175 phosphopeptide.

### pSer175 phosphorylation of CDK9 is a marker of activated P-TEFb in primary memory CD4^+^ T cells

**Figure 10:**
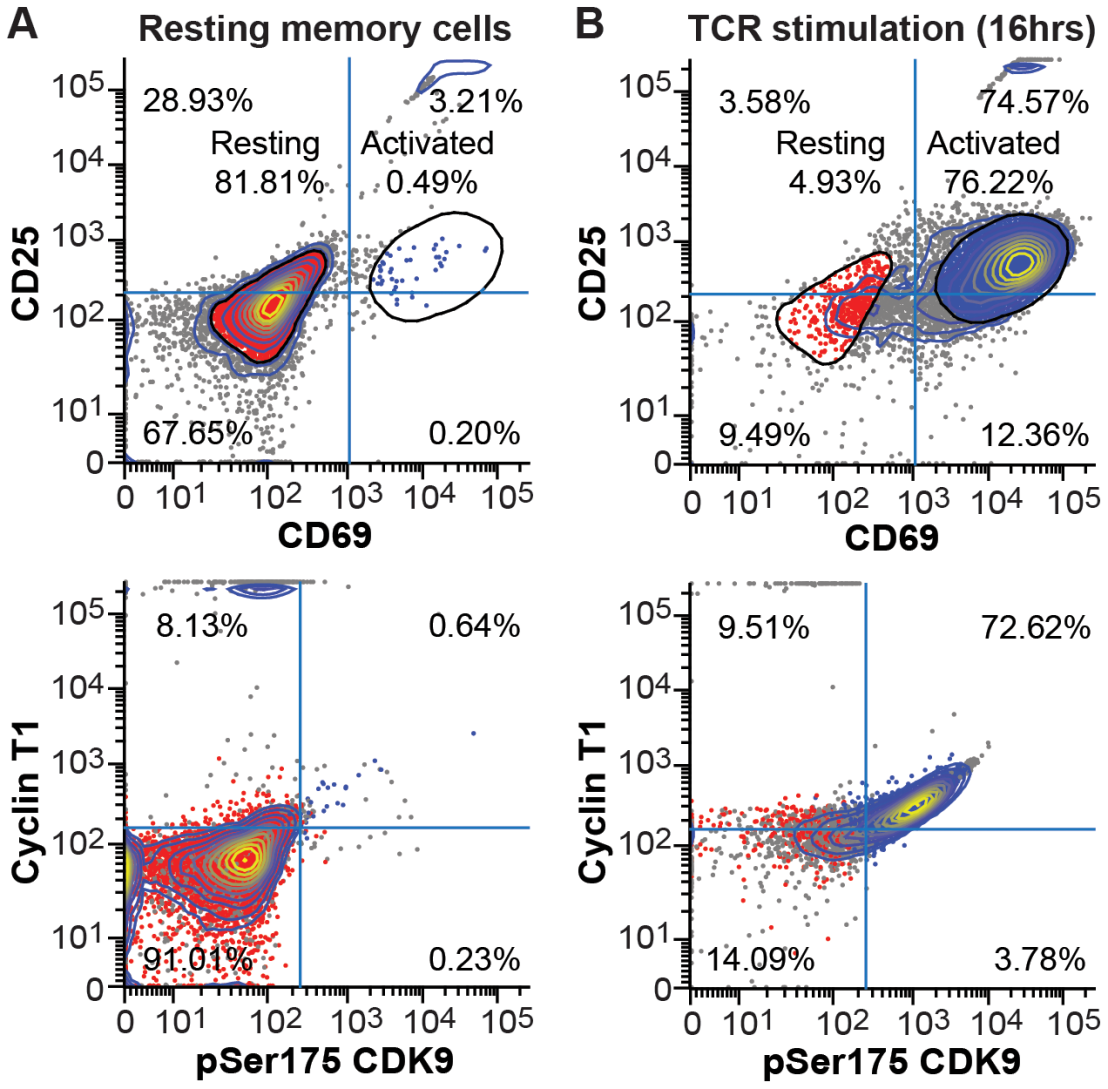
Activated (CD25^+^ CD69^+^) memory CD4^+^ T-cells have elevated expression of pSer175 CDK9 and CycT1. (A) Resting memory CD4^+^ T-cells isolated from a healthy donor stained for flourophore-conjugated antibodies against the T-cell activation markers (CD25 and CD69) and P-TEFb components (Cyclin T1 and pSer175 CDK9) components and then analyzed by multicolor flow cytometry. (B) Cells from the same donor activated for 16 hr with α-CD3 and α-CD28 mAbs.

To determine whether cells expressing high levels of pSer175 CDK9 also carried T-cell activation markers, memory CD4^+^ T cells were purified from healthy donor peripheral blood using negative bead selection and stimulated for 16 hr anti-CD3 and anti-CD28 antibodies to activate the TCR. These cells were analyzed by flow cytometry after staining with fluorophore-conjugated antibodies against pSer175 CDK9 and hCycT1 (**Fig. 10**). As expected, after stimulation through the TCR 76.22% of the resting cell population shifted to an activated memory CD4^+^ T cell phenotype (CD25^+^CD69^+^) (**Fig. 10B**). More than 95.5% of the activated cells showed significantly elevated expression of pSer175 CDK9 and hCycT1 (**Fig. 10B**). As shown in **Fig. S4B** the pSer175 peptide effectively blocked the pSer175 signal in the flow cytometry assay, again demonstrating the specificity of the antibody binding.

### Kinetics of pSer175 phosphorylation of CDK9 in response to T-cell receptor stimulation

**Figure 11:**
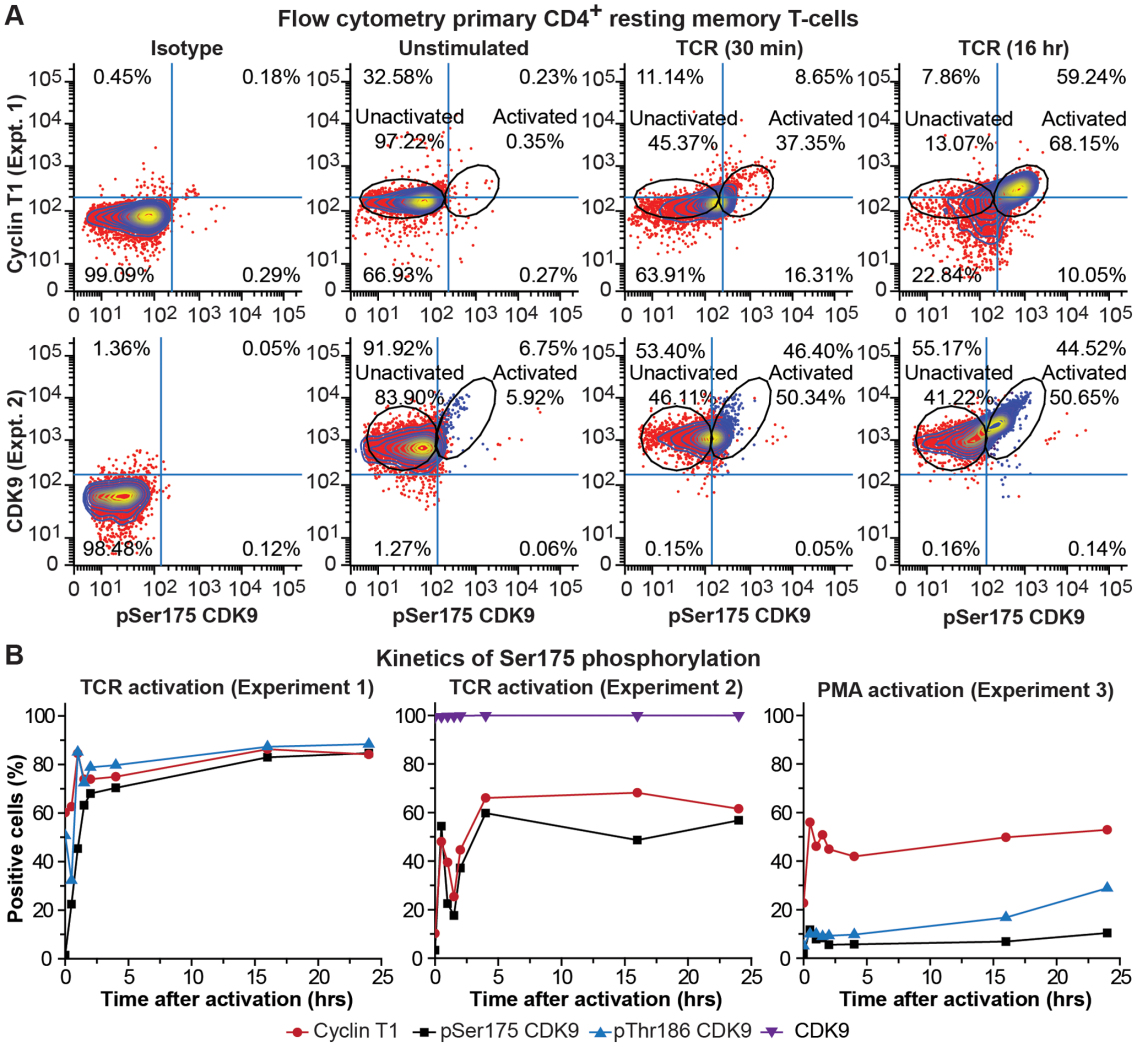
Rapid induction of Ser175 phosphorylation of CDK9 after stimulation of resting memory CD4+ T cells through the TCR. (A) Flow cytometry. Memory CD4^+^ T-cells isolated from healthy donors were stimulated for 2 hr or 24 hr with α-CD3 and α-CD28 mAbs to activate the TCR, immunostained with the flourophore-conjugated antibodies against total CDK9, pThr186 CDK9, CycT1 and pSer175 CDK9 and analyzed by multicolor flow cytometry. Top panels: Representative data from Experiment 1 (**Figs. S5 and S6**). Bottom panels: Representative data from Experiment 2 (**Figs. S7 and S8**). (B) Kinetic analysis. Time course of P-TEFb activation in primary resting memory CD4^+^ T-cells stimulated between 0 and 24 hrs with α-CD3 and α-CD28 mAbs or with PMA. Kinetic data from two TCR and one PMA activation (**Figs. S9 and S10**) experiments are shown. The fraction of positive cells staining for pThr186 (blue lines), CycT1 (red lines), pSer175 CDK9 (black lines), and total CDK9 (black lines) were measured by flow cytometry as shown in Panel A. Note that during the course of the experiment there is also a 2- to 10-fold increase in the mean fluorescent intensity for the CDK9 and CycT1 proteins that is not represented by the data for % positive cells.

The alterations in P-TEFb levels following TCR exposure are extremely rapid. As shown in **Fig. 11** and **Figs. S5 to S10**, the flow cytometric assay described above was used to monitor the kinetics of P-TEFb activation in resting memory T-cells. As shown in **Fig. 11A**, increases in CycT1 levels and pSer175 CDK can be detected as early as 30 min after stimulation through the TCR. A detailed kinetic analysis is shown in **Fig. 11B** based on the data shown in **Figs. S5 to S10**. In Experiment 1 (**Figs. S5, S6**), following TCR activation pSer175 CDK9 levels rose from undetectable (1.4% positive cells) to 70.3% positive cells during the first 4 hrs. During the next 20 hrs there was a gradual rise in the pSer175 CDK9^+^ cells reaching 84.7% at 24 hrs. There was a parallel rise in CycT1 and pThr186 levels, but because these markers were present at low, but measurable levels, a significant fraction of the resting cell population scored as a positive signal (60.1% CycT1 and 50.7% pThr186 CDK). Therefore, the use of these markers to define the activated cell phenotype is less reliable than the pSer175 marker.

Similar results were obtained in Experiment 2 ((**Figs. S7, S8**), where total CDK9, CycT1 and pSer175 CDK9 levels were monitored using flow cytometry. In this experiment essentially all the resting cells were positive for CDK9. However, total CDK9 levels rose gradually during the next 24 hrs, consistent with previous reports that total CDK9 expression is largely unaffected by TCR stimulation of resting CD4^+^ T cells [36], [72]. Therefore, the rapid induction of pSer175 could not be attributed to an elevation of CDK9 expression. In this experiment, approximately 10% of the resting cells were positive for CycT1 but less than 3% were positive for pSer175 CDK9. After TCR stimulation CycT1 and pSer175 CDK9 levels rapidly increased showing peaks at 30 min before reaching plateau levels by 4 hrs. The complex kinetics of P-TEFb induction seen in this experiment probably reflect fluctuations in TCR signaling due to the cyclical downregulation of the receptor [90], [91], [92].

We also examined P-TEFb responses to PMA stimulation of resting memory T-cells (Experiment 3, **Figs. S9, S10**). Compared to TCR stimulation, we consistent found that increases in pSer175 and pThr186 levels were comparatively small following PMA activation of the primary T-cells. This may help to explain relatively poor activation of latent HIV in resting memory cells by PMA [33], [93].

In Jurkat T-cells, PMA is a highly effective inducer of CDK9 Ser175 phosphorylation. Flow cytometry demonstrates that after 2 hr exposure to PMA, 18.8% of the Jurkat T-cells showed elevated pSer175 levels and 76.8% showed an elevation in RNAP II Ser2 C-terminal domain (pSer2-CTD) phosphorylation, a marker of P-TEFb phosphorylation of RNAP II (**Fig. S11**). Similar activation profiles were seen following 4 hr stimulation through the TCR, but TNF-α treatment did not elevate either pSer175 or pSer2-CTD levels.

## Discussion

### Regulation of P-TEFb activity by cellular signaling pathways

In recent studies using Jurkat T-cells we demonstrated that TCR signaling regulates P-TEFb activity [53]. Examination of P-TEFb complexes by gel filtration chromatography showed that both PMA and TCR signaling led to the rapid dissociation of the large inactive 7SK snRNP complex and release of lower molecular weight P-TEFb complexes. The disruption of the 7SK snRNP correlated with a global increase in the association of P-TEFb with chromatin, recruitment of P-TEFb to the HIV provirus and a rapid increase in HIV elongation. Both P-TEFb recruitment to the HIV LTR and enhanced HIV processivity were blocked by the ERK kinase inhibitor U0126 but not by AKT and PI3 kinase inhibitors. Thus, TCR signaling, mediated through the PKC and ERK kinase pathways provides the first example of a physiological pathway that can shift the balance between the inactive and active P-TEFb pools and thereby stimulate proviral reactivation.

The effect of TCR signaling on HIV transcription could only be demonstrated in latently infected cells carrying proviruses that encoded functional Tat genes. Although Tat is itself capable of serving as an activator of P-TEFb by physically extracting the kinase complex from 7SK snRNP [74], [75], [77], in latently infected cells this is not occurring to a measurable extent, suggesting that there is a critical threshold level of Tat that will allow it to compete with HEXIM1 for P-TEFb. We therefore hypothesized that the disruption of 7SK snRNP triggered by signal-dependent changes in posttranslational modifications of one or more of its protein components is able to increase the interactions between extremely low levels of presynthesized Tat and P-TEFb.

In the present study we employed AP-MS/MS using Jurkat T-cells stably expressing FLAG-CDK9 to define the molecular requirements for the signal-dependent dissociation of P-TEFb from 7SK snRNP and its enhanced interaction with Tat to mediate proviral transcription elongation. This approach successfully led to the isolation of all the known 7SK snRNP protein components, mapping of substantial portions of their amino acid sequences, and the identification of novel PTMs on each of these proteins (**Tables 1** **to** **5**). PTMs involving acetylation, methylation and phosphorylation of each of the protein components of the complex were identified, but relatively few of these modifications were enhanced in response to PMA or TCR signaling. The identified PTMs in **Tables 1** **to** **5** are based on data obtained in Jurkat T-cells which are actively dividing and have assembled 7SK RNP complexes. Many of these reported modifications are likely to be restricted in resting memory T-cells, as is the case for pThr186. We therefore expect that the majority of the modifications detected in Jurkat T-cells could only be present in activated primary T-cells. Although the identification of these modifications can be further strengthened using high resolution MS/MS data collection for site assignment, the tandem MS spectral quality of these modified peptide precursors was sufficient under the optimum (LTQ Velos ion trap) MS/MS data collection conditions to allow us to unambiguously make site assignments.

### Phosphorylation of Ser175 regulates the competition between Tat and BRD4 for P-TEFb binding

The phosphorylation event that showed the greatest increase in response to TCR or PMA signaling was the phosphorylation of CDK9 at Ser175, a highly conserved residue located in the activation loop of the kinase. In the Tat/P-TEFb X-ray structure published by Tahirov et al. [88], Ser175 is situated at the Tat/CDK9 interface about 6.5 Å away from Lys12 of Tat. Quantitative MS/MS analysis revealed that stimulation of Jurkat T cells with PMA, a potent activator of the MAPK/ERK pathway, induced >10-fold increase in Ser175 phosphorylation in three different experiments. We were able to confirm these MS/MS findings by pSer175 immunoblotting analysis of FLAG-CDK9 affinity purified complexes isolated from non-stimulated and PMA-treated cells. Ammosova et al. [79] also identified Ser175 as an *in vivo* phosphorylation site using Hunter peptide mapping of *in vivo* -labeled ^32^P-peptides followed by confirmation of the peptide sequence by mass spectroscopy.

Our structural models revealed that phosphorylation of CDK9 at Ser175 permits formation of a hydrogen bond with Lys12 of Tat and also strengthen intermolecular interactions postulated to exist between the activation loop of CDK9 and the N-terminal region of Tat. In support of this model is the observation that the S715A CDK9 mutation resulted in a 1.7-fold disruption of CDK9 association with Tat without interfering with CDK9/hCycT1 interaction while the phosphomimetic S175D CDK9 mutation resulted in a modest increase in Tat association (**Fig. 5**).

Mutations in S175 also had profound effects on interactions between CDK9 and BRD4. In agreement with the original studies of Yang et al. [80], who performed similar experiments using HeLa cells transfected with FLAG-tagged CDK9 plasmids, and Ammosova et al. [79], we found that S175A resulted in an almost complete block of CDK9 binding to BRD4 (1.9% of wildtype). However, we found in contrast to their results that S175D had 50% BRD4 binding capacity compared to wildtype. Therefore, we believe that BRD4 is probably also able to interact with Ser175-phosphorylated CDK9.

A P-TEFb interacting domain (PID) has been identified at the C-terminal end of BRD4 [94] and was shown to be sufficient to induce the dissociation of P-TEFb from 7SK snRNP [77], [95]. Overexpression of the PID alone in cells induced HEXIM1 and 7SK snRNA dissociation from P-TEFb, but it is not sufficient to activate Tat-independent transcription of the HIV LTR [95]. Although the region(s) of P-TEFb that interact with the PID of BRD4 have not been fully defined, our current observations suggest that the activation loop of CDK9 carrying phosphorylated Ser175 plays an important role in mediating P-TEFb interaction with BRD4.

### BRD4 restricts Tat binding to P-TEFb and helps to sustain proviral latency

Expression of CDK9 carrying mutations in Ser175 unexpectedly resulted in the activation of latent proviruses. The stable expression of the phosphomimetic mutation S175D CDK9 resulted in a 2.7-fold (E4 cells, wildtype Tat) and 3.7-fold (2D10 cells, H13L Tat) enhancement of HIV proviral gene expression (**Fig. 7**). These observations provide additional support for the idea that phosphorylation of Ser175 promotes a favorable interaction between CDK9 and Tat. However, the mutation with the strongest phenotype was S175A, which induced a 45-fold (E4 cells, wildtype Tat) and 20-fold (2D10 cells, H13L Tat) increase in HIV proviral gene expression. Since S175A reduced CDK9 association with Tat, we were surprised to observe that this mutation so effectively reactivated HIV proviral gene expression. We have attributed the S175A phenotype to elimination of the competition between Tat and BRD4 binding to P-TEFb for two reasons: a) S175A caused a much more severe disruption of CDK9 association with BRD4 than with Tat; and b) Stable knockdown of BRD4 expression in latently infected Jurkat T cells also led to a robust reactivation of proviral gene expression in a Tat-dependent manner [85], [86], [96]. Ammosova et al. [79] have also observed that expression of S175A is able to activate HIV transcription.

Transactivation of HIV by CDK9 mutants could only be observed when proviruses expressed functional Tat genes. This implies that BRD4 is not used to sustain basal HIV transcription in the absence of Tat. Instead, it appears likely that Tat and BRD4 compete for P-TEFb binding and that high basal BRD4 levels serve to restrict HIV transcription when Tat levels are low. Reductions in the levels of BRD4 in the cell, or reductions in the affinity of P-TEFb for BRD4, are therefore expected to permit extremely low levels of Tat to bind to P-TEFb and initiate HIV transcription.

Our model that phosphorylation of S175 enhances its interactions with Tat and promotes HIV transcription differs from the model of Ammosova et al. [79] who reported that dephosphorylation of Ser175 by PP1 upregulated HIV-1 transcription. However, since PP1 is a promiscuous enzyme, and CDK9 carrying unphosphorylated Ser175 is able to interact with Tat, it is possible that they have observed an indirect effect of the PP1 treatment rather than a phenotype directly ascribable to Ser175 dephosphorylation.

### pSer175 provides a sensitive marker for activated P-TEFb levels in primary T-cells

Resting memory CD4^+^ T-cells isolated from healthy donor peripheral blood were screened for Ser175 phosphorylation using a highly specific antibody exclusively recognizing CDK9 carrying pSer175. Using a novel flow cytometric assay, we found that resting memory CD4^+^ T-cells are highly restricted in hCyT1 expression and Thr186 phosphorylation of CDK9 (pThr186), in agreement with previous work from the Rice laboratory [36], [37], [38], [73].

Consistent with the results seen in Jurkat T-cells, unactivated primary cells also showed no detectable pSer175 CDK9. Stimulation of resting memory CD4+ T-cells through the TCR with α-CD3 and α-CD28 antibodies or by PMA resulted in a rapid elevation of hCycT1, and pThr186 CDK9 levels. Similar results were obtained using immunofluorescence, which also demonstrated that CDK9 carrying the pSer175 modification associates with nuclear speckles, a region believed to correspond to the site of active transcription. pSer175 levels rose with slightly delayed kinetics compared to that of hCycT1 expression and pThr186 and fluctuated substantially during a 24 hr time course while hCycT1 and pThr186 were relatively constant after 2 hr post-stimulation. The fluctuating pSer175 levels are probably a reflection of fluctuations in TCR receptor signaling due to the cyclical downregulation of the receptor [90], [91], [92]. Thus, pSer175 phosphorylation represents a separate and specific step in the activation of p-TEFb. Similar results were obtained using immunofluorescence, which also demonstrated that CDK9 carrying the pSer175 modification associates with nuclear speckles, a region believed to correspond to sites of active transcription [73].

We are currently adapting this flow assay to monitor the activation state of T-cells in clinical studies for the evaluation of compounds that reactivate HIV as part of the “shock and kill” strategy for viral eradication. Preliminary results in collaboration with Dr. David Margolis have shown that both pThr186 and pSer175 levels rise in CD4^+^ T-cells recovered from patients exposed to SAHA.

**Figure 12:**
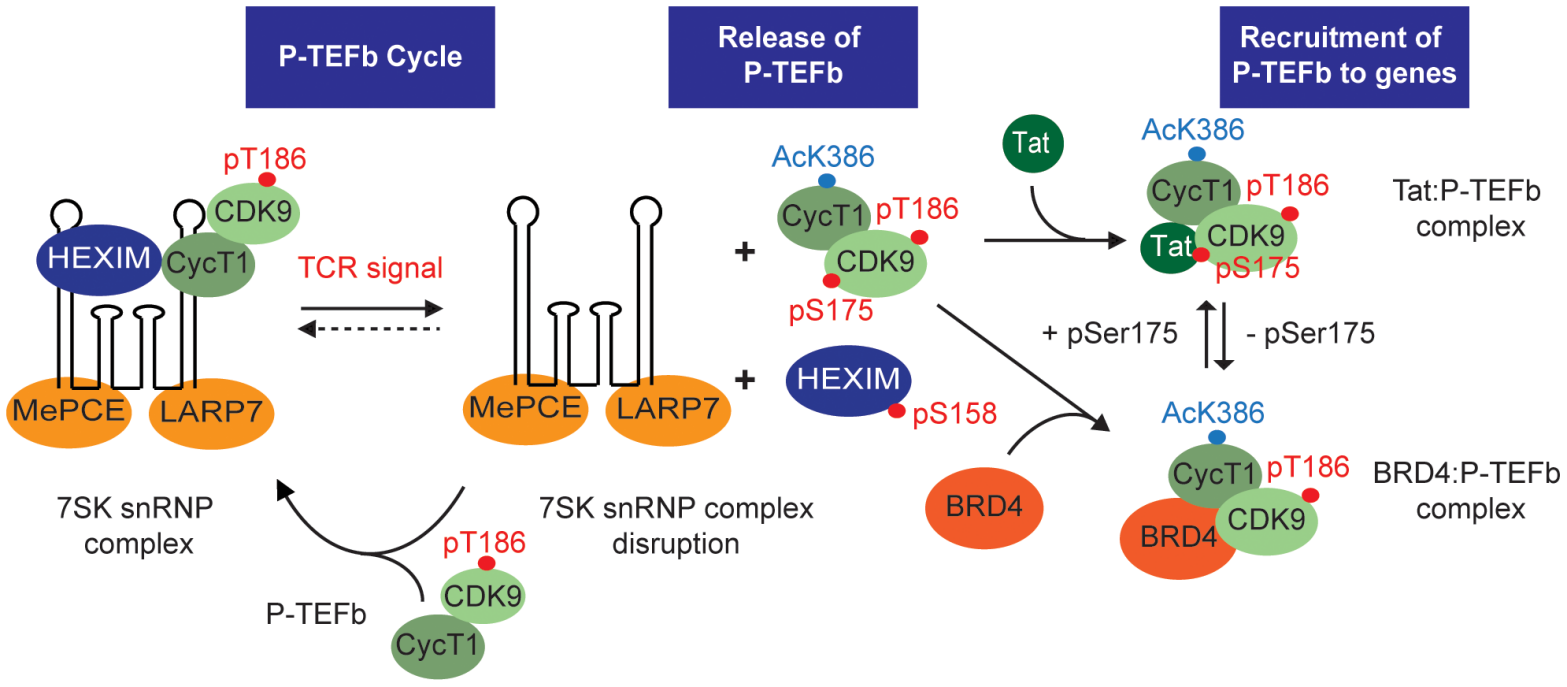
Model for the activation of P-TEFb in memory CD4^+^ T-cells. In resting memory CD4^+^ T-cells P-TEFb expression is restricted due to low levels of CycT1. T-cell receptor activation of the cells leads to new CycT1 synthesis, phosphorylation of CDK9 at Thr186, and the assembly of the transcriptionally inactive 7SK snRNP complex containing 7SK RNA, HEXIM1 and the RNA binding proteins MePCE and LARP7. TCR signaling disrupts the 7SK RNP complex and leads to phosphorylation of CDK9 on Ser175 (this paper), HEXIM1 on Ser158 [78], and acetylation of CycT1 on K386 [8181] which shift the binding equilibrium away from the 7SK RNP complex. Tat and BRD4 compete for P-TEFb binding with the equilibrium being shifted in favor of Tat binding when Ser175 is phosphorylated.

The flow cytometry and immunofluorescence data are consistent with a model where the initial incorporation of P-TEFb into 7SK snRNP requires hCycT1 and pThr186 but not pSer175 (**Fig. 12**). Consistent with this we found that CDK9 phosphorylated at Ser175 is absent from complexes purified using tagged-HEXIM1, which is highly enriched for the 7SK snRNP complex. Furthermore, the T186A and T186D mutants of CDK9 which cannot be incorporated into 7SK snRNP are modified at Ser175 in a signal-dependent manner. Finally, U0126, which is a potent inhibitor of MAPK/ERK signal-dependent disruption of the 7SK snRNP complex [53], only modestly inhibited formation of pSer175, suggesting that it is unable to block phosphorylation of the pre-existing “free” P-TEFb. Thus we conclude that Ser175 phosphorylation is probably not a primary signal leading to the disruption of the 7SK snRNP complex, but is instead a modification that is introduced subsequent to the liberation of P-TEFb from 7SK snRNP. The separate acquisition of the pThr186 and pSer175 modifications during T-cell activation is also consistent with the idea that pSer175 is absent from the inactive 7SK snRNP complex. It is interesting to speculate that modifying CDK9 at Ser175 not only enhances its interactions with BRD4 and Tat but also inhibits the reassociation of P-TEFb with HEXIM1 and 7SK snRNP. Follow-up studies are underway to identify the kinase that is responsible for pSer175 in order to precisely define the P-TEFb activation pathway.

In addition to the phosphorylation of CDK9 Ser175 that we have reported here, Fujinaga et al. [78] have recently reported that HEXIM1 is also phosphorylated in response to TCR signaling. Specifically they showed that there is a protein kinase C (PKC)-dependent phosphorylation of HEXIM1 on Ser158, a site believed to be within the RNA-binding domain of HEXIM1. Direct binding experiments showed that the phosphorylated HEXIM1 protein has a reduced affinity for 7SK snRNA and is unable to inhibit the enzymatic activity of P-TEFb [78].

Thus, it seems likely that multiple post-synthetic modifications of P-TEFb and 7SK RNP components are used to regulate P-TEFb assembly and disassembly. Besides the phosphorylation of CDK9 Ser175 and HEXIM1 Ser158, there are acetylation and methylation events that take place on CDK9, CycT1 and HEXIM1. Cho et al. [81] have reported that acetylation of CycT1 on 4 residues (K380, K386, K390 and K404) triggers dissociation of the 7SK snRNA complex and activates the transcriptional activity of P-TEFb. Consistent with their results we found that acetylation of K386 was enhanced almost 2-fold after PMA stimulation. Additional modifications we are currently evaluating for their potential functional significance include, acetylation and methylation of CDK9 Lys127, and acetylation of HEXIM1 Lys284.

In conclusion the activation of P-TEFb in these cells is a multi-step process that involves the initial assembly of P-TEFb into 7SK snRNP, signal-dependent release of P-TEFb from this inactive complex, and the phosphorylation of CDK9 at Ser175. In this paper, we have identified pSer175 as a modification at the activation loop of CDK9 that plays a critical role in altering the competitive binding of Tat and BRD4 to P-TEFb. pSer175 also provides an easily detected molecular marker for the transcriptionally active form of P-TEFb in primary CD4^+^ T-cells.

## Materials and Methods

### Materials

RPMI 1640 medium and fetal bovine serum were purchased from Hyclone. PMA and LARP7 antibody were purchased from Sigma. U0126 was obtained from Calbiochem. α-CD3 and α-CD28 antibodies were obtained from BD Biosciences. CDK9, hCycT1, and TRITC-conjugated hCycT1 antibodies were from Santa Cruz Biotechnology. Phospho-Thr186 CDK9 antibody was purchased from Cell Signaling Technology. HEXIM1 and BRD4 antibodies were custom synthesized and affinity purified by Covance Research Products. The MSCV retroviral expression system (Clontech) was used in the current study for the creation of stable Jurkat T-cell lines expressing FLAG-CDK9 wt, the indicated FLAG-CDK9 point mutants, or FLAG-HEXIM1.

### Generation of the phospho-Ser175 CDK9 antibody

Rabbit polyclonal antiserum towards a 19-residue phospho-Ser175 epitope for CDK9 (ADFGLARAFpSLAKNSQPNR) was generated at Covance Research Products. After affinity isolation of the antibody using the phospho-serine peptide, eluted antibody fractions were subjected to multiple rounds of purification by negative selection with affinity resin for the corresponding unmodified peptide. Ser175Ala and Ser175Asp mutants of CDK9 were used to confirm by Western blotting that the purified antibody was selective towards the phospho-Ser175 epitope.

### Large scale affinity purification and FLAG-CDK9 and FLAG-HEXIM1 complexes

8.0×10^8^ Jurkat T cells stably expressing FLAG-CDK9 or FLAG-HEXIM1 were treated or not with 50 ng/mL PMA or 25 µM DRB for 1 h. After washing the cells twice with 1× PBS, whole cell extracts (WCEs) were prepared using cell lysis buffer A [150 mM NaCl, 10 mM KCl, 1.5 mM MgCl_2_, 0.5% NP-40, 1 mM DTT, 10 mM Hepes pH 8.0] or RIPA buffer [150 mM NaCl, 0.5% Triton X-100, 0.5% Sodium deoxycholate, 0.1% SDS, 5 mM EDTA, 20 mM Tris HCL, pH 7.5] containing a cocktail of protease and phosphatase inhibitors. WCEs were cleared by centrifugation at 2000 rpm for 5 min and 2 h incubation with protein A sepharose beads prior to incubating them overnight at 4°C with anti-FLAG M2 agarose beads (Sigma). Immunoprecipitates were washed extensively with cell lysis buffer A or RIPA buffer and elution of FLAG-CDK9 or FLAG-HEXIM1 complexes was performed by overnight incubation at 4°C with 200 µg/mL FLAG peptide (Sigma) in buffer A or RIPA buffer.

### Sample preparation for LC-MS/MS

FLAG peptide protein eluates were concentrated by methanol-chloroform precipitation and rehydrated with 1× LDS loading buffer containing 50 mM DTT before being resolved by 1D SDS-PAGE on a 4–12% Bis-Tris gel. After SDS-PAGE the gel was stained with SYPRO Ruby (Invitrogen) as recommended by the manufacturer. Protein bands on the gel were visualized on a UV light source whose surface had been cleaned with 50% isopropanol. Selected bands were excised from the gel with a sterile blade, crushed in 50 mM ammonium bicarbonate buffer, pH 8, before being subjected to a standard in-gel digest protocol involving a reduction step with 20 mM DTT and alkylation with 55 mM iodoacetamide in the dark. Reduction was preceded by wetting the gel pieces to its brim with 50 mM Ammonium bicarbonate buffer (pH 8.0) for 15 minutes followed by addition of 50% acetonotrile/25 mM Ammonium bicarbonate buffer for 15 minutes. Appropriate washing step of adding 50 mM Ammonium bicarbonate buffer (50 mM) was also done in between the reduction and alkylation steps. An extra wash was done just before drying the gel pieces in 100% acetronitrile. After removal of the organic solvent followed by drying of the gel pieces, overnight tryptic digestion step was performed at 37°C with the addition of 200 ng of sequencing grade trypsin (Promega Inc, WI) in 50 µL of 50 mM ammonium bicarbonate buffer, pH 8. Afterwards, 0.2% formic acid was added to stop the proteolytic processing and the resulting peptides were extracted from the supernatant fraction of the in-gel digest along with the recovered fractions from 2 rounds of 50% acetonitrile/0.3% formic acid extractions. Upon drying and resolubilization in 25 µL of 0.1% formic acid, the samples were processed for the LC-MS/MS analysis as described below.

### LC-MS/MS analysis

The digests prepared above were analyzed by LC-MS/MS using a Waters nano Acquity UPLC system (Waters Inc, MA) that was interfaced to a LTQ Velos-Orbitrap mass spectrometer (Thermo-Finnigan, Bremen, Germany). The platform was operated in the nano-LC mode using the standard nano-ESI API stack fitted with a picotip emitter (uncoated fitting, 10 µm spray orifice, New Objective, Inc., Woburn, MA). The solvent flow rate through the column was maintained at 300 nL/min using the split-free Acquity system. The protein digests (7 µL) were injected into a reversed-phase symmetry C18 trapping column (0.18×20 mm, 5 µm particle size, Waters Inc.) equilibrated with 0.1% formic acid (FA)/2% acetonitrile (v/v) and washed for 5 min with the equilibration solvent at a flow rate of 15 µL/min, using the sample trapping mode of UPLC. After the washing step, the trapping column was switched in-line with a reversed-phase C18 nanoacquity UPLC column (0.075×250 mm, Waters Inc.) and the peptides were separated using a linear gradient of acetonitrile from 5% to 45% in aqueous 0.1% formic acid over a period of 60 min (0.67% gradient) at the above-mentioned flow rate such that the eluate was directly introduced to the mass spectrometer. A 100% acetonitrile elution step was subsequently performed for 10 minutes prior to resetting the analytical column to the initial equilibration conditions for 15 more minutes at the end of the chromatographic run, making a total run time of 90 min. for the LC-MS/MS analysis. The mass spectrometer was operated in a data-dependent MS to MS/MS switching mode, with the 10 most intense ions in each MS scan subjected to MS/MS analysis. The full scan was performed at 60000 resolution in the Orbitrap detector and the MS/MS fragmentation scans were performed in the Velos dual ion trap detector (IT) CID mode. The threshold intensity for the MS/MS trigger was always set at 1000 and the fragmentation was carried out using the CID mode using a normalized collision energy (NCE) of 35. The data was entirely collected in the profile mode for the full scan and centroid mode for the MS/MS scans. Dynamic exclusion function for previously selected precursor ions was enabled during the analysis such that the following parameters were applied: repeat count of 2, repeat duration of 45 seconds, exclusion duration of 60 seconds and exclusion size list of 450. Xcalibur software (version 2.0.7), Thermo-Finnigan Inc., San Jose, CA) was used for instrument control, data acquisition, and data processing.

### Mass spectrometry data analysis

LC MS/MS files exported from XCalibur were directly used for the protein database searching step. For all the search tasks, Mascot was the preferred search engine with the public IPI human (Version June 2010) database. Search parameter file for probing S/T/Y phosphorylation included the following settings: precursor mass tolerance of 5 ppm, fragment mass tolerance of 0.8 Da, 1 missed cleavage, use of decoy database and post-translational modification (PTM) search options including 1) cysteine carbamidomethylation, 2) methionine oxidation and 3) S,T/Y phosphorylation. In case of the database search for alternate PTMs such as methylation and acetylation, separate searches were initiated on the same raw files that included 1) Methylation (R,K) 2) Dimethyl (R,K) 3) Acetylation (K) apart from Cysteine carbamidomethylation as possible modifications to be probed at the same time. Protein identification were subject to strict data QC filtering within MASCOT at peptide level such as (a) peptide expectation value of 0.05 or less and b) single peptide hits being ignored whereas the peptide identifications made any for post-translation modifications (PTMs) were subject to manual verification using an extended MS/MS interpretation routine that accommodates potential neutral losses in the mass spectrometer collision cell (CID mode).

For quantitative data analysis to determine the abundance of the selected peptides of interest in control and treated samples using a relative peptide quantitation approach, in which the modified and unmodified forms of the peptide were successfully identified , the 325–1800 m/z full-scan, MS1, high resolution MS data was used to perform the extracted ion chromatogram (XIC) analysis based on a label-free quantitation method that relies on chromatographic peak area calculation after peak smoothing. This process was done manually since there were only a few precursor masses of interest in this study. In the case of CDK9's T186 containing-peptide 179-NSQPNRYTNR-188, the T186 site was detected only in the phosphorylated form and hence the relative abundance of this peptide under different conditions was calculated with reference to an internal standard-like peptide 295-LLVLDPAQR-303 using the commonly accepted abundant peptide ratiometric approach [97]. To maintain consistency in our quantitation approach, the relative quantitation for the S175 phosphorylation event was also performed using the detected 173-AFSLAK-178 peptide with reference to the 295-LLVLDPAQR-303 abundant peptide ratiometrically even though both the modified and unmodified forms of the 173-AFSLAK-178 were detected most of the time and that the peak areas of the S175-phosphorylated/modified and unmodified forms can be used independently to make a determination of their relative abundances under different conditions of interest. To calculate the variance of quantitation encountered using the XIC analysis, n = 3 data collection was performed for S175 phosphorylation and the data analysis procedure followed the method described above with the exception that abundance peptide approach was applied only if needed and that the relative peptide approach was the preferred approach.

### Analysis of WCEs and anti-FLAG or anti-HA immunoprecipitates by Western blotting

Jurkat T cells were engineered to stably express FLAG-CDK9, FLAG-CDK9 mutants or FLAG-HEXIM1. Cells were maintained in RPMI 1640 medium supplemented with 5% fetal bovine serum, penicillin (100 IU/mL), streptomycin (100 µg/mL), and 25 mM Hepes at 37°C in 5% CO_2_. After the appropriate experimental treatments as discussed in the Results section, WCEs were prepared using cell lysis buffer A and cleared by microcentrifugation at 5000 rpm for 5 min. Protein concentration of WCEs was determined using the BCA protein assay kit (Pierce) and equal amounts of WCEs were resolved by NuPAGE 1D-SDS PAGE (Invitrogen). Following transfer of resolved proteins to a nitrocellulose or PVDF membrane, Western blotting analysis was performed with antibodies against CDK9, HEXIM1, Cyclin T1, LARP7, phospho-Thr186 CDK9, phospho-Ser175 CDK9, Tat, FLAG, and HA epitopes.

Cleared WCEs were also subjected to immunoprecipitation with anti-FLAG M2 agarose beads or anti-HA agarose beads (Sigma). After extensive washes of the immunoprecipitates with cell lysis buffer A, protein elution was performed with 200 µg/mL of either FLAG or HA peptide, and the eluates were analyzed by Western blotting as discussed above.

### Radioactive *in vitro* kinase assays

Whole cell extracts were prepared from Jurkat T cells (5×10^8^) belonging to uninfected, CDK9 wt, S175A, S175D, T186A, and T186D and used to immunopurify FLAG-CDK9 complexes using anti-FLAG M2 affinity beads (Sigma). The *in vitro* kinase reaction was set up as follows with or without the addition of 250 ng His-tagged full length human RNAP II CTD repeat substrate (Abcam): 20 µL of the anti-FLAG protein immune complex in kinase dilution buffer (50 mM Hepes, pH 7.5, 1 mM DTT), 2 µCi of γ^32^ ATP, 1.5 µM ATP, and 25 µL of 2× Standard assay buffer (100 mM Hepes, pH 7.5, 2 mM DTT, 6 mM MgCl_2_, 6 mM MnCl_2_) containing a phosphatase inhibitor cocktail. The kinase reaction was performed at 30°C for 1 h. Thereafter, reactions were stopped by boiling the samples in LDS sample loading buffer, subjected to SDS-PAGE on a 4–12% Bis-Tris gel (Invitrogen), and the dried gel was analyzed by autoradiography.

### Isolation of CD4^+^ T cells and immunoflourescence staining for flow cytometry

Heparinized peripheral blood was obtained from a healthy donor and used to isolate peripheral blood mononuclear cells (PBMCs). Memory CD4^+^ T cells were purified from PBMCs using negative bead selection and challenged with a combination of anti-CD3 and anti-CD28 antibodies to activate the T-cell receptor. For surface staining with anti-CD25 and anti-CD69, cells were first washed with autoMACS buffer (Miltenyi Biotech) and then incubated with each fluorochrome-conjugated antibody for 30 min in the dark and on ice. For intracellular staining with anti-Cyclin T1, anti-phospho-Ser175 CDK9, and anti-phospho-Thr186 CDK9, surface stained cells were washed with autoMACS buffer and permeabilized and fixed using the Foxp3 Fixation/Permeabilization Kit (eBioscience) according to the manufacturer's protocol. Thereafter, these cells were rinsed twice with 1× permeabilization buffer resuspended in 1× permeabilization buffer containing 2% mouse serum, and incubated for 15 min at room temperature. Anti-phospho-Ser175 CDK9, and anti-phospho-Thr186 CDK9 antibodies were conjugated using Zenon labeling kit (Invitrogen) immediately prior to use. Fluorochrome-conjugated antibodies were added to the cells and incubation was performed for 30 min in the dark and on ice. After rinsing the cells twice with 1× Permeabilization buffer they were subjected to flow cytometry analysis using the LSR Fortessa instrument equipped with a red 640 laser with a 780/60 and 670/30 filter.

### Immunofluorescence for microscopic analysis

Memory CD4^+^ T cells were allowed to adhere to cover slips coated with poly-L-lysine for 5 min at 37°C. The cells were fixed and permabilized for 30 minutes using the FoxP3 Fixation/Permabilization kit (eBioscience). Afterwards the cells were blocked using 2% normal mouse IgG (The Jackson Laboratory) for 15 min. Antibodies for anti-phospho-Ser175 CDK9, anti-phospho-Thr186 CDK9, anti-SC35 Nuclear Speckle Marker (Abcam), anti-Cyclin T1(Santa Cruz Biotechnologies) and DAPI were added for 15 min. Cells were washed three times and Cy5, Cy2 secondary antibodies were added for 15 min and subsequently washed three times. The cover slips were mounted using gel mount (Electron Microscope Sciences) and viewed using a DeltaVision epifluorescent microscope (Applied Precision). Images were captured in *z* series, deconvolved, and processed using the Softworx analysis program (Applied Precision). Images were exported as TIFF or JPEG files, and Figures were composed using Adobe Photoshop CS.

### Structural modeling studies using the Tat/P-TEFb crystal structure as template

The 2.1 Å X-ray structure of HIV-1 Tat complexed with human P-TEFb was retrieved from protein data bank (PDB ID: 3MIA). A model was prepared for the phosphorylated serine at position 175 of CDK9. The structural conformation of this modified Tat/P-TEFb complex was energy minimized to remove any unfavorable contacts and to allow for productive interactions which might occur as a result of the modification. Energy minimization was performed in explicit solvent environment by solvating the structures in TIP3P waters. Two-step energy minimization was performed – first only the water molecules were energetically relaxed keeping the protein fixed and in the second step the whole system was energy minimized without any restraints. Each step involved minimization employing combination of steepest descent and conjugate gradient algorithms. AMBER 11 suite of programs was used perform the calculations using FF99SB force field. Parameters for phosphorylated serine were obtained from AMBER parameter database.

### Ethics statement

Procedures for obtaining anonymous blood donations from healthy volunteers were approved by the University Hospitals of Cleveland IRB (Number: 12-11-33). Adult healthy volunteers were recruited by the Case CFAR Clinical Core from the immediate community by flyer advertisements. The only procedure that the volunteers (healthy nonpregnant adults who weigh at least 110 pounds) underwent was venipuncture to obtain peripheral blood samples. This was performed by trained phlebotomists in the Case CFAR Clinical Core. Verbal and written consent was obtained from adult volunteers participating in this study by persons who have been certified in human subject protection regulations. Blood samples were assigned a code number unrelated to the volunteer's medical record number.

## Supporting Information

Figure S1 **A representative Mascot database search result of CDK9 isolated from the Flag-CDK9 immunoprecipitates of untreated Jurkat 2D10 cell lines.** Top: Matched peptides for CDK9 isoform 1. Bottom: Mascot data showing the peptides detected for CDK9 isoform 1along with their precursor masses and mass accuracies of the peptides detected. The data has been filtered for MASCOT expectation score of 0.05 and for the requirement that all peptides designated to be unique in their identity for CDK9. Positive identification of unmodified and phosphorylated S175 is observed as part of the AFSLAK peptide detection.
(TIFF)

Figure S2 **XIC calculations for S175 modifications.** Top: A representative total ion current chromatographic profile at the base peak level as well as sample extracted ion chromatograms (XIC) are shown for a control Flag-CDK9 gel band digest's sample (Test A2) . Bottom: Relative phosphorylation at the S175 site is inferred using the relative peptide quantitation approach as detailed in the Materials and Methods section of the manuscript.
(TIF)

Figure S3 **MS/MS (tandem MS) fragmentation spectra (FT-FT MS/MS Data collection).** To unambiguously validate the FT-IT AP-MS proteomics data, commercially synthesized modified and unmodified S175 containing hexapeptides were subjected to FT- FT MS/MS CID fragmentation experimentation. 2 (A) Representative high resolution tandem MS spectrum for unmodified AFSLAK synthetic peptide. (B) phosphorylated AFSLAK peptide. High accuracy fragment mass detection provides validation to the FT-IT discovery data.
(TIF)

Figure S4 **Validation of the epitope specificity of the pSer175 CDK9 antibody for immunostaining and flow cytometry analysis by peptide blocking.** Resting memory T-cells and T-cells activated for 16 hr by anti-CD3 and anti-CD28 antibodies were stained with fluorophore conjugated antibodies against pSer175 CDK9 and total CDK9. For the peptide blocking experiments, the purified antibody was pre-incubated overnight with phospho-Ser175 peptide. (A) Immunofluorescence. (B) Flow cytometry.
(TIF)

Figures S5 **Kinetic analysis of P-TEFb activation in memory CD4^+^ T-cells: Cyclin T1 versus pSer175 CDK9 (TCR Activation, experiment 1).** Resting memory CD4^+^ T-cells isolated from a healthy donor were stimulated for with α-CD3 and α-CD28 mAbs to activate the TCR and analyzed by multicolor flow cytometry. Samples were analyzed at 0, 0.5, 1, 1.5, 2, 4, 6, 16 and 24 hr after activation. Cells were stained with fluorophore conjugated antibodies towards CycT1 (vertical axis) and pSer175 CDK9 (horizontal axis). Quantititative analyses of these data (using a gating strategy to detect individual proteins) are shown in **Fig. 11B**.
(TIF)

Figures S6 **Kinetic analysis of P-TEFb activation in memory CD4^+^ T-cells: pT186 CDK9 versus pSer175 CDK9 (TCR Activation, experiment 1).** Resting memory CD4^+^ T-cells isolated from a healthy donor were stimulated for with α-CD3 and α-CD28 mAbs to activate the TCR and analyzed by multicolor flow cytometry. Samples were analyzed at 0, 0.5, 1, 1.5, 2, 4, 6, 16 and 24 hr after activation. Cells were stained with fluorophore conjugated antibodies towards pThr186 CDK9 (vertical axis) and pSer175 CDK9 (horizontal axis). Quantititative analyses of these data (using a gating strategy to detect individual proteins) are shown in **Fig. 11B**.
(TIF)

Figures S7 **Kinetic analysis of P-TEFb activation in memory CD4^+^ T-cells: Total CDK9 versus pSer175 CDK9 (TCR Activation, experiment 2).** Resting memory CD4+ T-cells isolated from a healthy donor were stimulated for with α-CD3 and α-CD28 to activate the TCR and analyzed by multicolor flow cytometry. Samples were analyzed at 0, 0.5, 1, 1.5, 2, 4, 6, 16 and 24 hr after activation. Cells were stained with fluorophore conjugated antibodies towards total CDK9 (vertical axis) and pSer175 CDK9 (horizontal axis). Quantititative analyses of these data (using a gating strategy to detect individual proteins) are shown in **Fig. 11B**.
(TIF)

Figure S8 **Kinetic analysis of P-TEFb activation in memory CD4^+^ T-cells: Cyclin T1 versus pSer175 CDK9 (TCR Activation, experiment 2).** Resting memory CD4+ T-cells isolated from a healthy donor were stimulated for with α-CD3 and α-CD28 to activate the TCR and analyzed by multicolor flow cytometry. Samples were analyzed at 0, 0.5, 1, 1.5, 2, 4, 6, 16 and 24 hr after activation. Cells were stained with fluorophore conjugated antibodies towards total CycT1 (vertical axis) and pSer175 CDK9 (horizontal axis). Quantititative analyses of these data (using a gating strategy to detect individual proteins) are shown in **Fig. 11B**.
(TIF)

Figure S9 **Kinetic analysis of P-TEFb activation in memory CD4+ T-cells: CycT1 versus pSer175 CDK9 (PMA Activation, experiment 3).** Resting memory CD4+ T-cells isolated from a healthy donor were stimulated for with 50 ng/mL PMA and analyzed by multicolor flow cytometry. Samples were analyzed at 0, 0.5, 1, 1.5, 2, 4, 6, 16 and 24 hr after activation. Cells were stained with fluorophore conjugated antibodies towards CycT1 (vertical axis) and pSer175 CDK9 (horizontal axis). Quantititative analyses of these data (using a gating strategy to detect individual proteins) are shown in **Fig. 11B**.
(TIF)

Figure S10 **Kinetic analysis of P-TEFb activation in memory CD4+ T-cells: pThr186 CDK9 versus pSer175 CDK9 (PMA Activation, experiment 3).** Resting memory CD4+ T-cells isolated from a healthy donor were stimulated for with 50 ng/mL PMA and analyzed by multicolor flow cytometry. Samples were analyzed at 0, 0.5, 1, 1.5, 2, 4, 6, 16 and 24 hr after activation. Cells were stained with fluorophore conjugated antibodies towards pThr186 CDK9 (vertical axis) and pSer175 CDK9 (horizontal axis). Quantititative analyses of these data (using a gating strategy to detect individual proteins) are shown in **Fig. 11B**.
(TIF)

Figure S11 **Induction of Ser175 phosphorylation by PMA stimulation of Jurkat T-cells coincides with 7SK snRNP dissociation and Ser2 CTD phosphorylation of RNAP II.** (A) Kinetics of Ser175 phosphorylation after PMA stimulation. Jurkat T-cells carrying FLAG-tagged CDK9 were induced by PMA and P-TEFb complexes were immunoprecipitated. Western blotting analysis (left) was performed on the anti-FLAG-CDK9 immunoprecipitates using antibodies against pT186 CDK9 (black line), pSer175 (red line) and LARP7 (green line). Data was normalized to total CDK9 levels detected in the immunoprecipitated samples. (B) Co-induction of pSer175-CDK9 and RNAP II CTD-pSer2. Jurkat 2D10 cells stably expressing FLAG-CDK9 were treated for the indicated times with PMA, TNF-α, or a combination of α-CD3 and α-CD28 antibodies to activate the TCR and analyzed by flow cytometry. Cells were stained with fluorophore-conjugated antibodies Alex Fluor 750-anti-pSer175 CDK9 and Alexa Fluor 647-anti-pSer2 RNAP II.
(TIF)
