# Supplementary figures and images for: Phosphorylation of CDK9 at Ser175 Enhances HIV Transcription and Is a Marker of Activated P-TEFb in CD4+ T Lymphocytes
Source: PLoS Pathog. 2013 May 2;9(5):e1003338. doi: 10.1371/journal.ppat.1003338 (PMC3642088; doi:10.1371/journal.ppat.1003338)

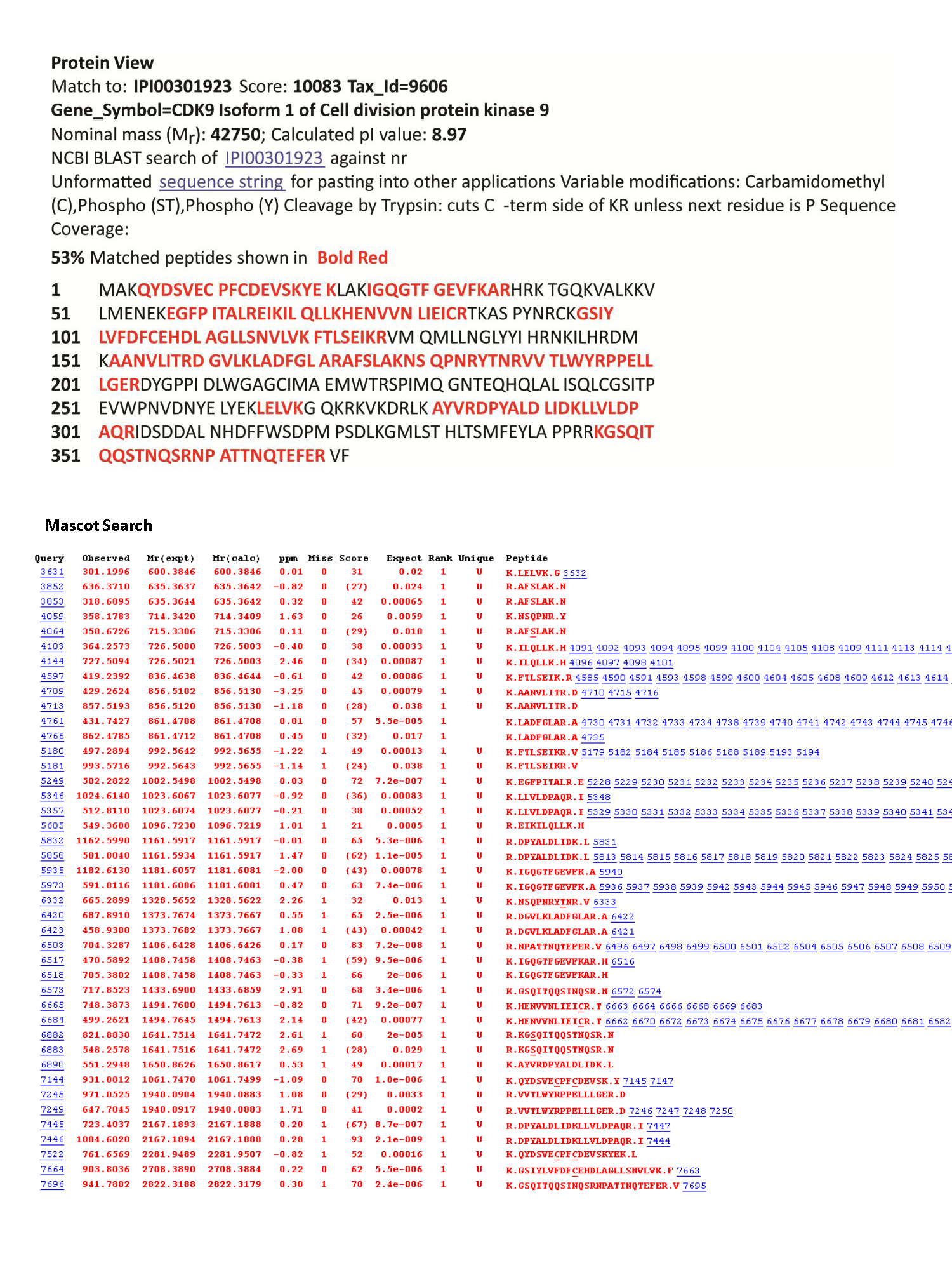

Supplement: Figure S1 — A representative Mascot database search result of CDK9 isolated from the Flag-CDK9 immunoprecipitates of untreated Jurkat 2D10 cell lines. Top: Matched peptides for CDK9 isoform 1. Bottom: Mascot data showing the peptides detected for CDK9 isoform 1along with their precursor masses and mass accuracies of the peptides detected. The data has been filtered for MASCOT expectation score of 0.05 and for the requirement that all peptides designated to be unique in their identity for CDK9. Positive identification of unmodified and phosphorylated S175 is observed as part of the AFSLAK peptide detection. (TIFF) [file ppat.1003338.s001.tiff]

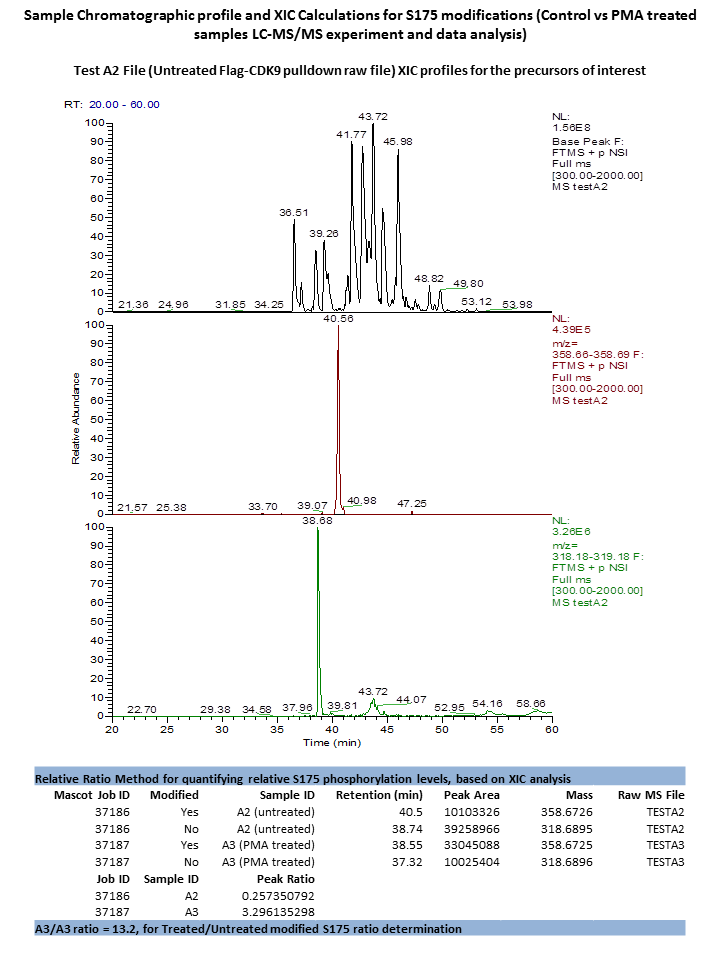

Supplement: Figure S2 — XIC calculations for S175 modifications. Top: A representative total ion current chromatographic profile at the base peak level as well as sample extracted ion chromatograms (XIC) are shown for a control Flag-CDK9 gel band digest's sample (Test A2) . Bottom: Relative phosphorylation at the S175 site is inferred using the relative peptide quantitation approach as detailed in the Materials and Methods section of the manuscript. (TIF) [file ppat.1003338.s002.tif]

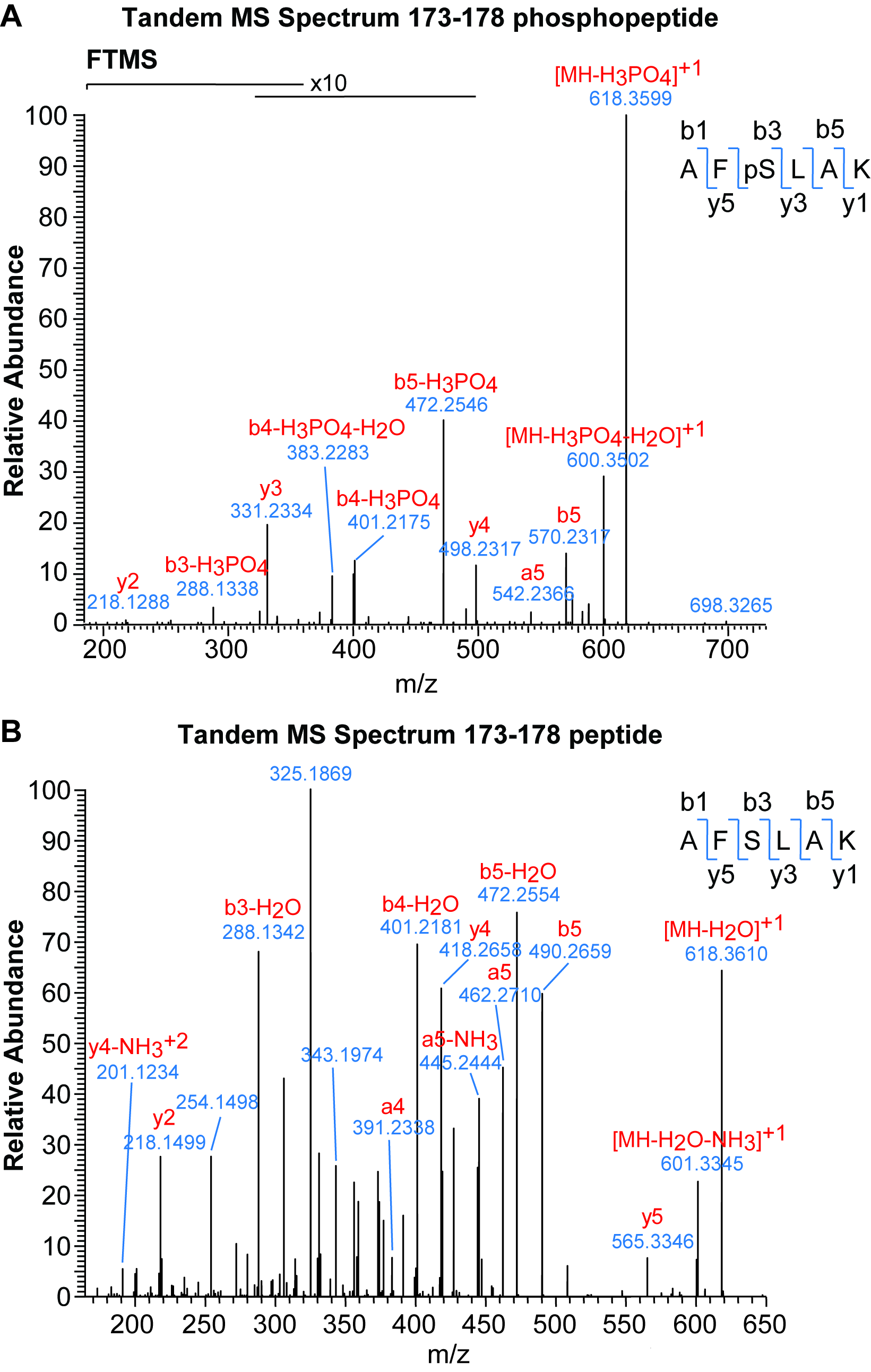

Supplement: Figure S3 — MS/MS (tandem MS) fragmentation spectra (FT-FT MS/MS Data collection). To unambiguously validate the FT-IT AP-MS proteomics data, commercially synthesized modified and unmodified S175 containing hexapeptides were subjected to FT- FT MS/MS CID fragmentation experimentation. 2 (A) Representative high resolution tandem MS spectrum for unmodified AFSLAK synthetic peptide. (B) phosphorylated AFSLAK peptide. High accuracy fragment mass detection provides validation to the FT-IT discovery data. (TIF) [file ppat.1003338.s003.tif]

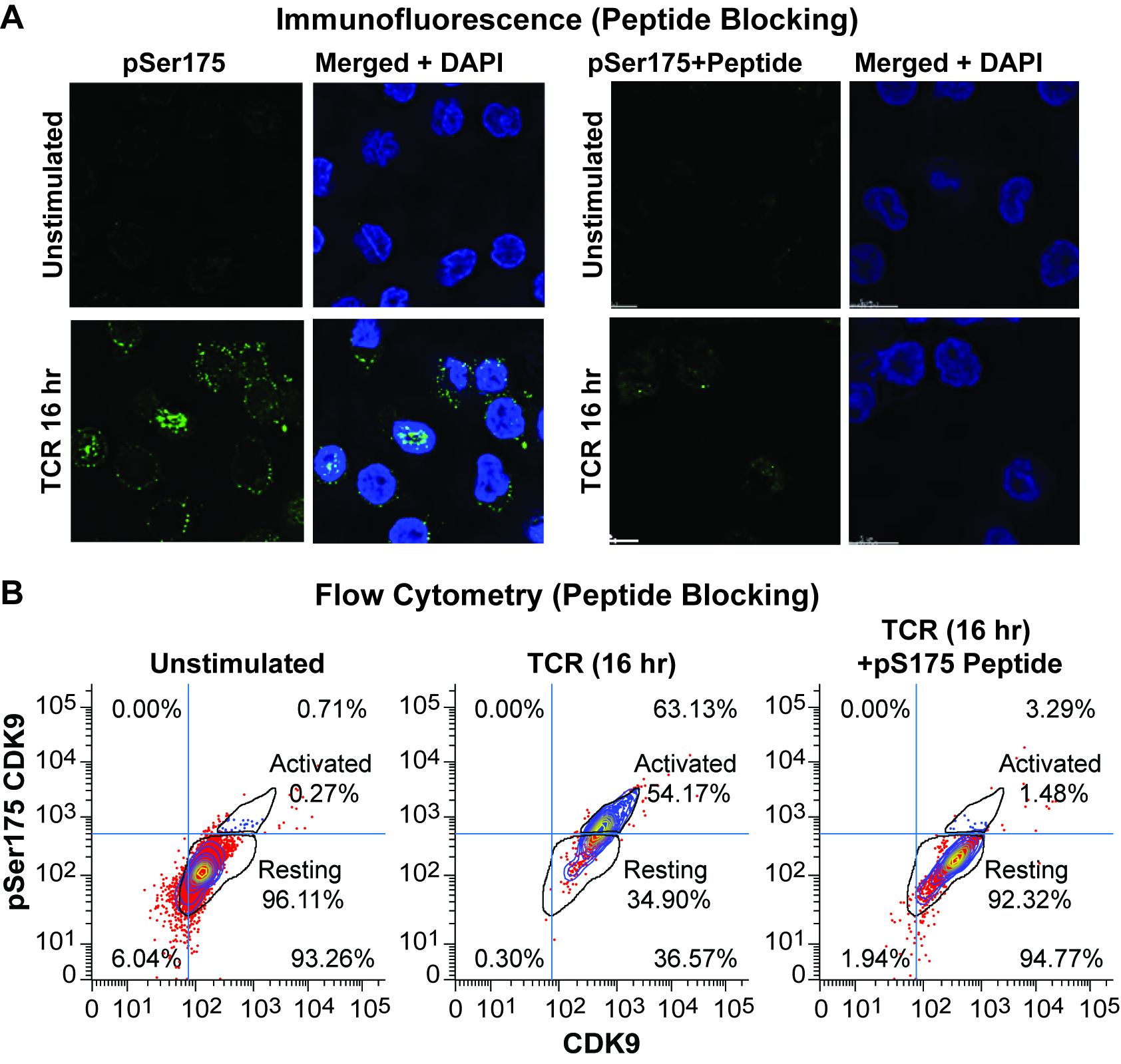

Supplement: Figure S4 — Validation of the epitope specificity of the pSer175 CDK9 antibody for immunostaining and flow cytometry analysis by peptide blocking. Resting memory T-cells and T-cells activated for 16 hr by anti-CD3 and anti-CD28 antibodies were stained with fluorophore conjugated antibodies against pSer175 CDK9 and total CDK9. For the peptide blocking experiments, the purified antibody was pre-incubated overnight with phospho-Ser175 peptide. (A) Immunofluorescence. (B) Flow cytometry. (TIF) [file ppat.1003338.s004.tif]

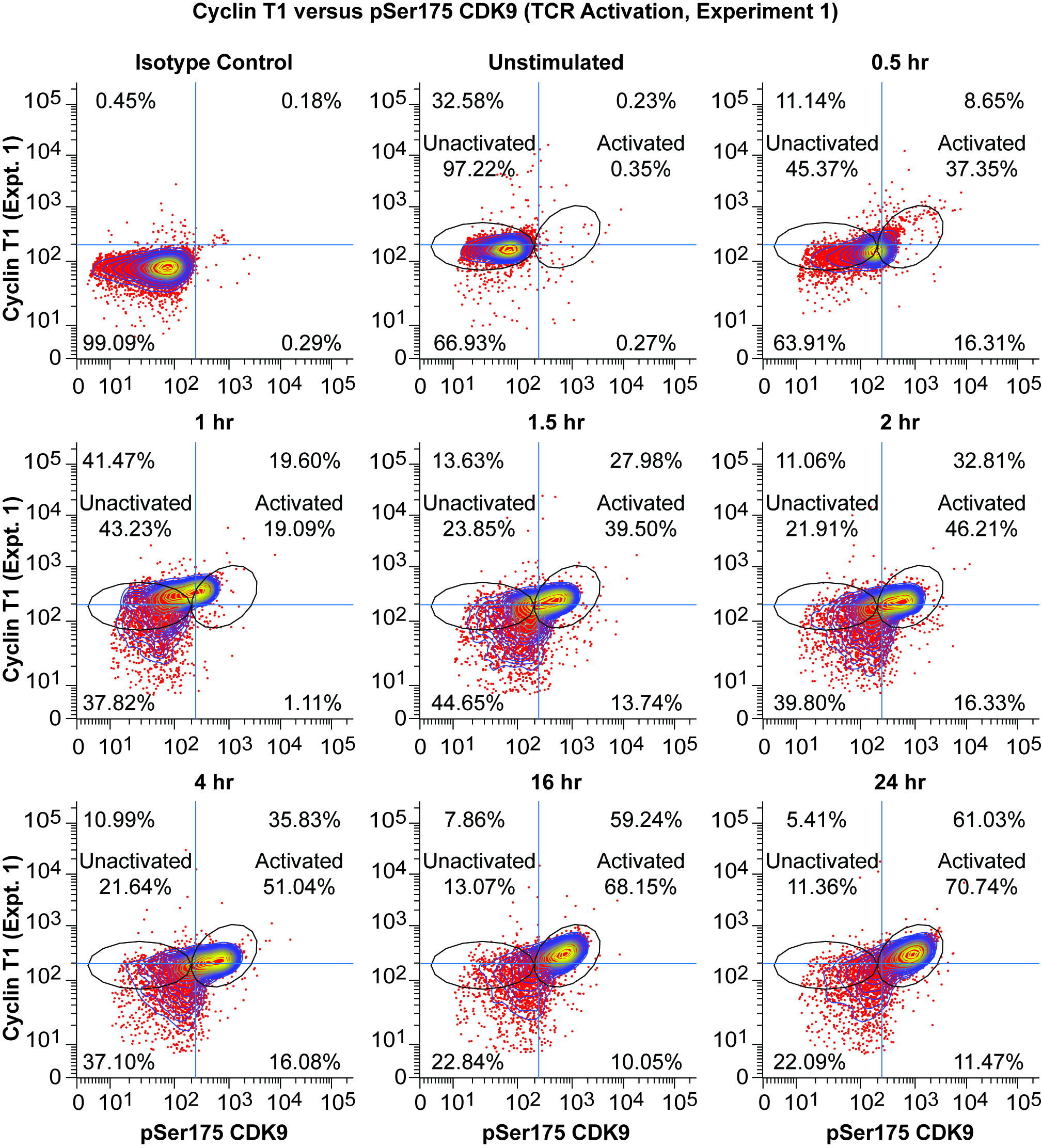

Supplement: Figures S5 — Kinetic analysis of P-TEFb activation in memory CD4+ T-cells: Cyclin T1 versus pSer175 CDK9 (TCR Activation, experiment 1). Resting memory CD4+ T-cells isolated from a healthy donor were stimulated for with α-CD3 and α-CD28 mAbs to activate the TCR and analyzed by multicolor flow cytometry. Samples were analyzed at 0, 0.5, 1, 1.5, 2, 4, 6, 16 and 24 hr after activation. Cells were stained with fluorophore conjugated antibodies towards CycT1 (vertical axis) and pSer175 CDK9 (horizontal axis). Quantititative analyses of these data (using a gating strategy to detect individual proteins) are shown in Fig. 11B . (TIF) [file ppat.1003338.s005.tif]

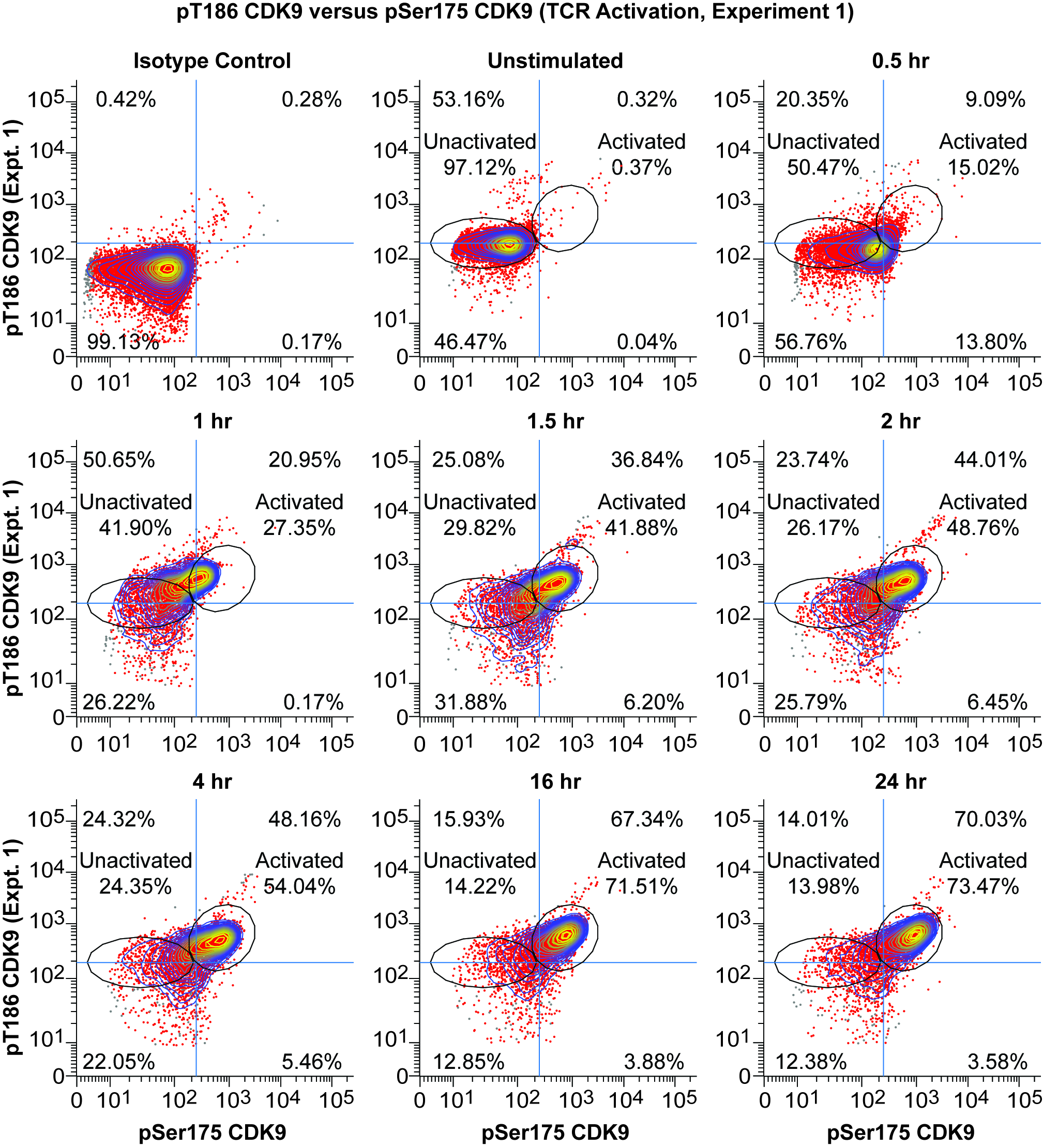

Supplement: Figures S6 — Kinetic analysis of P-TEFb activation in memory CD4+ T-cells: pT186 CDK9 versus pSer175 CDK9 (TCR Activation, experiment 1). Resting memory CD4+ T-cells isolated from a healthy donor were stimulated for with α-CD3 and α-CD28 mAbs to activate the TCR and analyzed by multicolor flow cytometry. Samples were analyzed at 0, 0.5, 1, 1.5, 2, 4, 6, 16 and 24 hr after activation. Cells were stained with fluorophore conjugated antibodies towards pThr186 CDK9 (vertical axis) and pSer175 CDK9 (horizontal axis). Quantititative analyses of these data (using a gating strategy to detect individual proteins) are shown in Fig. 11B . (TIF) [file ppat.1003338.s006.tif]

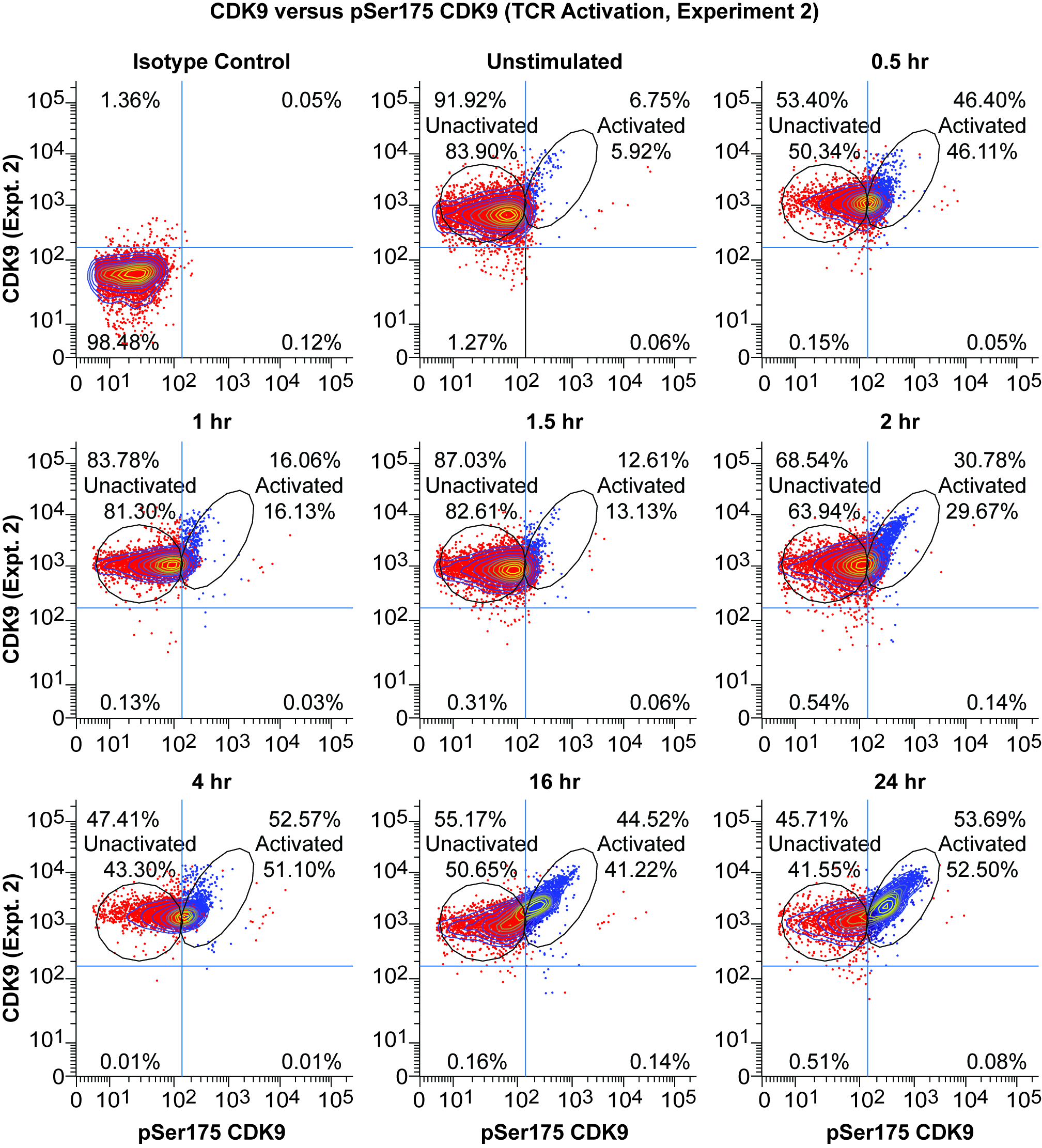

Supplement: Figures S7 — Kinetic analysis of P-TEFb activation in memory CD4+ T-cells: Total CDK9 versus pSer175 CDK9 (TCR Activation, experiment 2). Resting memory CD4+ T-cells isolated from a healthy donor were stimulated for with α-CD3 and α-CD28 to activate the TCR and analyzed by multicolor flow cytometry. Samples were analyzed at 0, 0.5, 1, 1.5, 2, 4, 6, 16 and 24 hr after activation. Cells were stained with fluorophore conjugated antibodies towards total CDK9 (vertical axis) and pSer175 CDK9 (horizontal axis). Quantititative analyses of these data (using a gating strategy to detect individual proteins) are shown in Fig. 11B . (TIF) [file ppat.1003338.s007.tif]

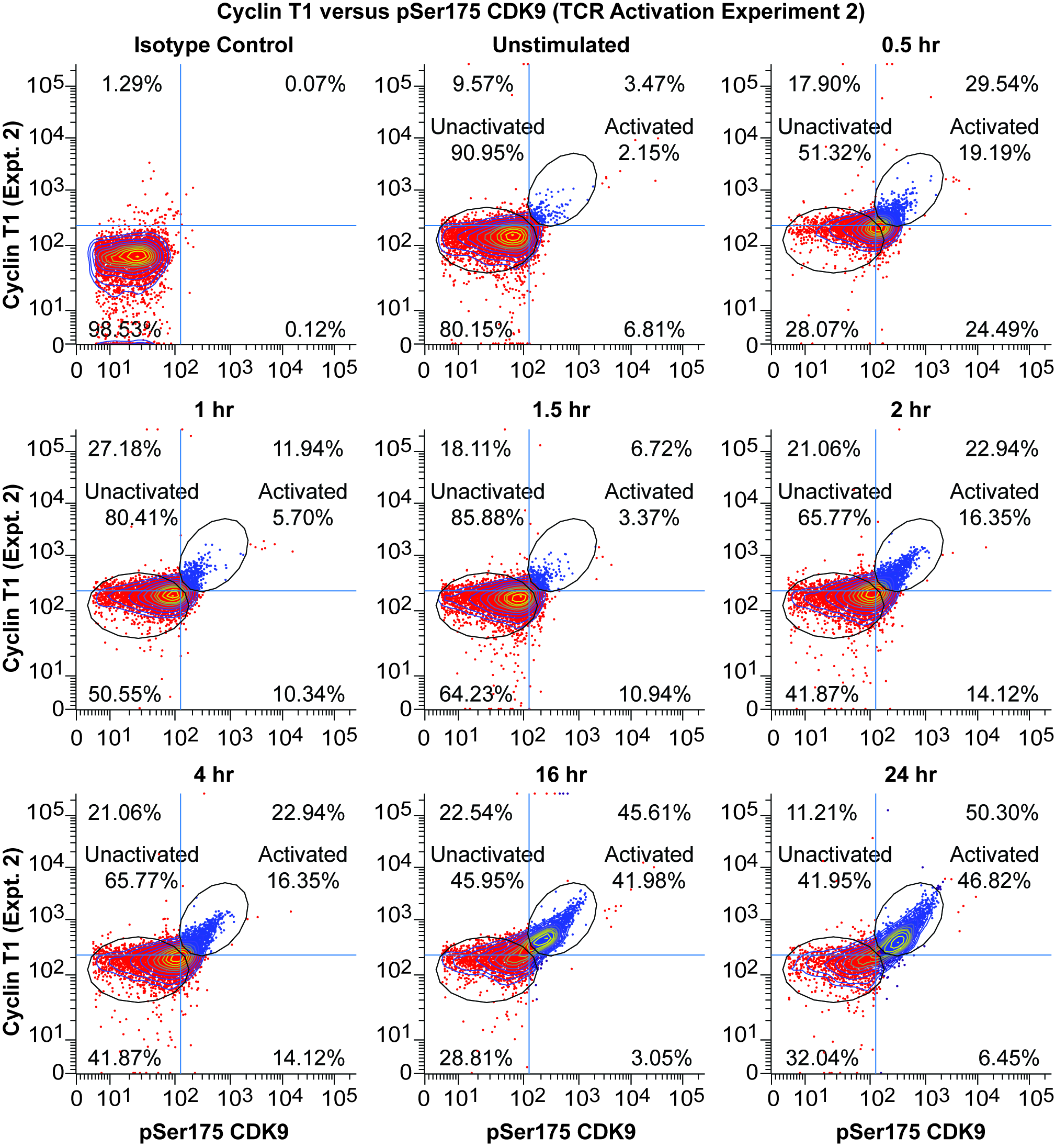

Supplement: Figure S8 — Kinetic analysis of P-TEFb activation in memory CD4+ T-cells: Cyclin T1 versus pSer175 CDK9 (TCR Activation, experiment 2). Resting memory CD4+ T-cells isolated from a healthy donor were stimulated for with α-CD3 and α-CD28 to activate the TCR and analyzed by multicolor flow cytometry. Samples were analyzed at 0, 0.5, 1, 1.5, 2, 4, 6, 16 and 24 hr after activation. Cells were stained with fluorophore conjugated antibodies towards total CycT1 (vertical axis) and pSer175 CDK9 (horizontal axis). Quantititative analyses of these data (using a gating strategy to detect individual proteins) are shown in Fig. 11B . (TIF) [file ppat.1003338.s008.tif]

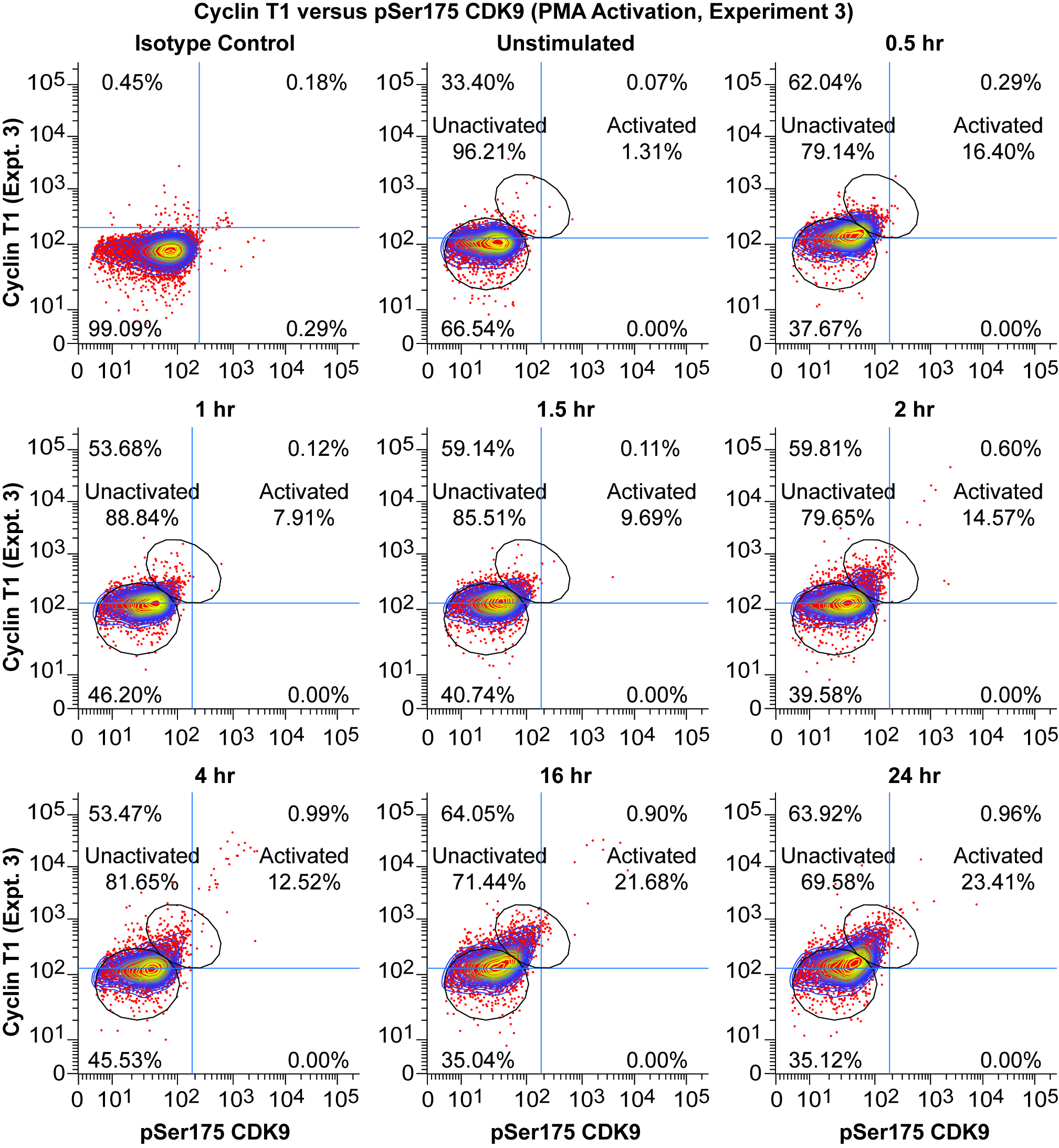

Supplement: Figure S9 — Kinetic analysis of P-TEFb activation in memory CD4+ T-cells: CycT1 versus pSer175 CDK9 (PMA Activation, experiment 3). Resting memory CD4+ T-cells isolated from a healthy donor were stimulated for with 50 ng/mL PMA and analyzed by multicolor flow cytometry. Samples were analyzed at 0, 0.5, 1, 1.5, 2, 4, 6, 16 and 24 hr after activation. Cells were stained with fluorophore conjugated antibodies towards CycT1 (vertical axis) and pSer175 CDK9 (horizontal axis). Quantititative analyses of these data (using a gating strategy to detect individual proteins) are shown in Fig. 11B . (TIF) [file ppat.1003338.s009.tif]

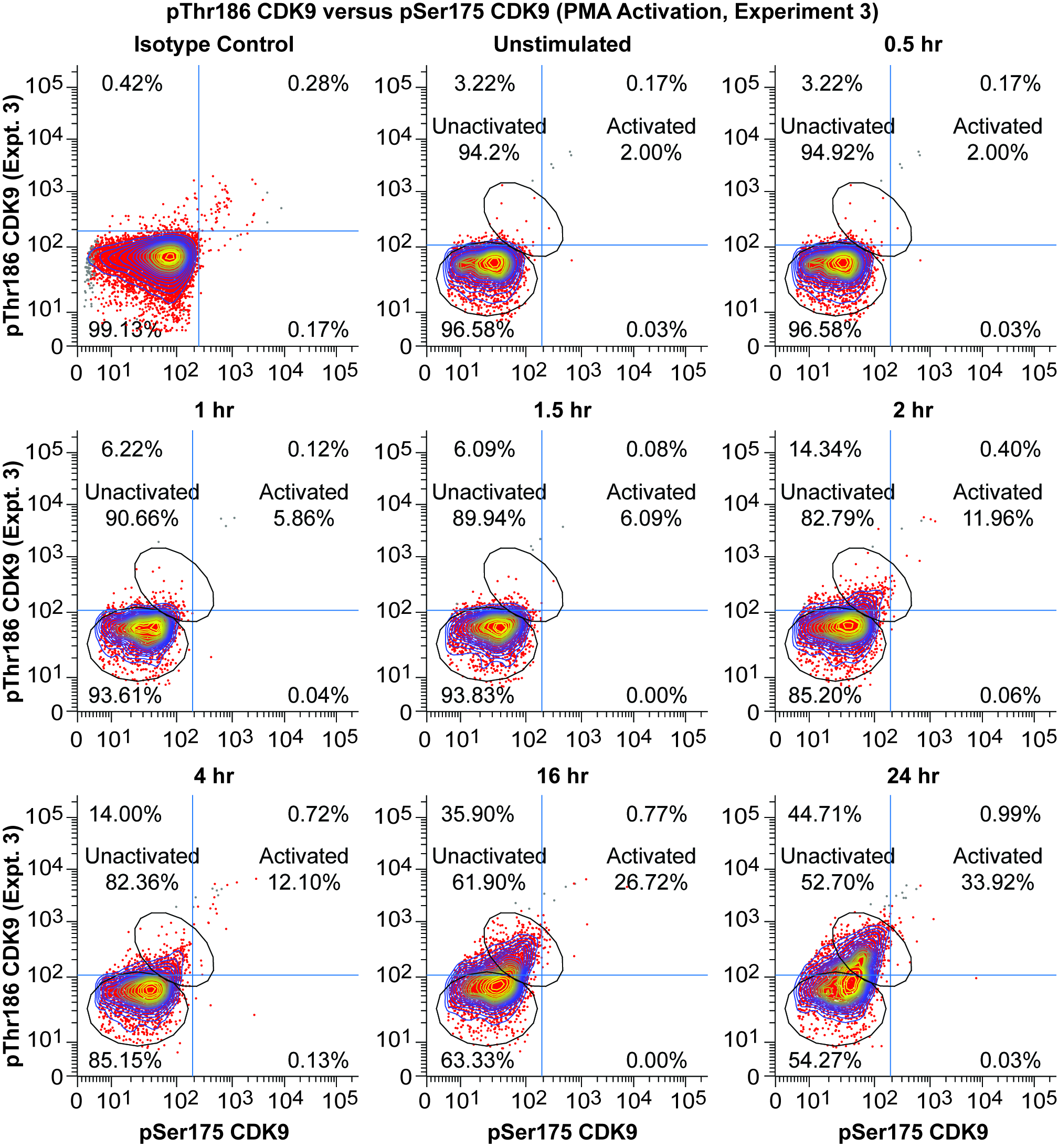

Supplement: Figure S10 — Kinetic analysis of P-TEFb activation in memory CD4+ T-cells: pThr186 CDK9 versus pSer175 CDK9 (PMA Activation, experiment 3). Resting memory CD4+ T-cells isolated from a healthy donor were stimulated for with 50 ng/mL PMA and analyzed by multicolor flow cytometry. Samples were analyzed at 0, 0.5, 1, 1.5, 2, 4, 6, 16 and 24 hr after activation. Cells were stained with fluorophore conjugated antibodies towards pThr186 CDK9 (vertical axis) and pSer175 CDK9 (horizontal axis). Quantititative analyses of these data (using a gating strategy to detect individual proteins) are shown in Fig. 11B . (TIF) [file ppat.1003338.s010.tif]

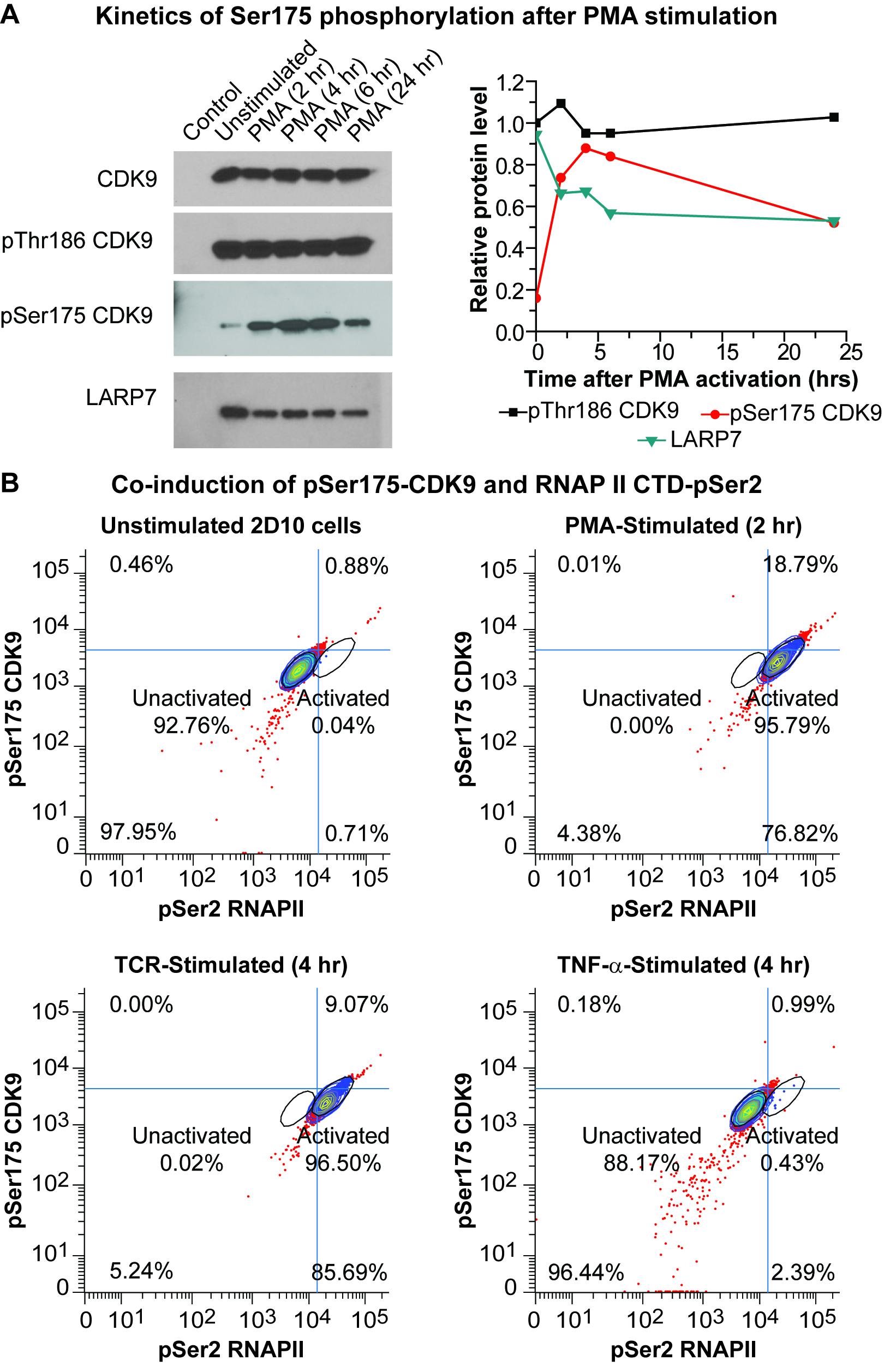

Supplement: Figure S11 — Induction of Ser175 phosphorylation by PMA stimulation of Jurkat T-cells coincides with 7SK snRNP dissociation and Ser2 CTD phosphorylation of RNAP II. (A) Kinetics of Ser175 phosphorylation after PMA stimulation. Jurkat T-cells carrying FLAG-tagged CDK9 were induced by PMA and P-TEFb complexes were immunoprecipitated. Western blotting analysis (left) was performed on the anti-FLAG-CDK9 immunoprecipitates using antibodies against pT186 CDK9 (black line), pSer175 (red line) and LARP7 (green line). Data was normalized to total CDK9 levels detected in the immunoprecipitated samples. (B) Co-induction of pSer175-CDK9 and RNAP II CTD-pSer2. Jurkat 2D10 cells stably expressing FLAG-CDK9 were treated for the indicated times with PMA, TNF-α, or a combination of α-CD3 and α-CD28 antibodies to activate the TCR and analyzed by flow cytometry. Cells were stained with fluorophore-conjugated antibodies Alex Fluor 750-anti-pSer175 CDK9 and Alexa Fluor 647-anti-pSer2 RNAP II. (TIF) [file ppat.1003338.s011.tif]
